# Migration extent and potential economic impact of the fall armyworm in Europe

**DOI:** 10.1038/s41598-025-02595-7

**Published:** 2025-05-19

**Authors:** Stelios Kartakis, Kiran J. Horrocks, Kutay Cingiz, Darren J. Kriticos, Justus Wesseler

**Affiliations:** 1 Agricultural Economics and Rural Policy Group, Wageningen University, Wageningen, The Netherlands; 2 Biosafety Research Group, Agroscope, Zürich, Switzerland; 3 Cervantes Agritech Pty Limited, Canberra, ACT Australia; 4 Applied Biosciences, Macquarie University, Sydney, NSW Australia

**Keywords:** Invasive species, CLIMEX, *Spodoptera frugiperda*, Partial budgeting, Pest invasion, Invasive species, Ecological modelling, Environmental economics, Invasive species

## Abstract

The fall armyworm (FAW), *Spodoptera frugiperda* (J.E. Smith, 1979), is a major agricultural pest native to the Americas that feeds on several crops, particularly maize. Since 2016, FAW has invaded more than 50 African countries, much of Southeast Asia, and Oceania, severely impacting agriculture and posing a threat to global food security. Following its recent detections in Europe in 2023, this study investigates FAW’s migratory capacity and potential direct economic impact on European grain maize production under a “no-control” scenario—areas previously unexplored in the context of Europe. We explored FAW’s potential distribution across the European continent by incorporating updated climatic data, refined parameter values, and an expanded occurrence dataset into a revised CLIMEX niche model. Our results reveal sizable potential economic impacts with the southern European Member States facing up to €546 ha^− 1^ grain maize gross margin annual losses, exceeding €900 million under the worst-case scenario. These member states bear the highest risk since they include areas projected to be climatically suitable for both permanent FAW establishment and transient populations during warmer months. This study provides important insights into the risks posed by FAW to Europe and can inform preparedness and decision-making to mitigate the economic consequences of its invasion.

**Supplementary Information:**

The online version contains supplementary material available at 10.1038/s41598-025-02595-7.

## Introduction

The fall armyworm (FAW), *Spodoptera frugiperda* (J.E. Smith, 1979) (Lepidoptera: Noctuidae), is a highly polyphagous pest that feeds on over 350 plant species from 76 botanical families^1,2^. Its larvae feed upon the aerial parts of these plants, causing severe yield losses in a wide range of economically important crops^2^. Notable among these are cereals, forage crops, and grasses, with a particular impact on maize, rice, and sorghum. Other crops affected include soybean, cotton, grapes, citrus, and berries^3–6^.

A wide host range, high reproductive potential, and strong migratory capacity contribute to FAW’s ability to invade new areas^7,8^. Native to the Americas, FAW has long been recognized as a major pest of maize, dating back to the 15th century^9,10^. In 2016, it was first detected outside its native range in West and Central Africa^11^ and within two years, it had spread throughout sub-Saharan Africa^2^. By 2018, it had been reported in India, Yemen, Bangladesh, Myanmar, Sri Lanka, and Thailand, and in 2019, it was detected in China, South Korea, and Japan^12^. Currently, FAW’s presence has been documented beyond its native range in 50 African countries and many parts of Southeast Asia and Oceania^12^. FAW’s rapid global emergence has led to its recognition as a threat to food production and security^13–15^. Additionally, several reports have demonstrated its resistance to conventional synthetic insecticides and transgenic *Bacillus thuringiensis* (Bt) crops^16–19^; methods conventionally employed for the control of this pest, which exacerbates the potential for FAW populations to inflict devastating yield losses.

Yield losses due to FAW vary across crops and management strategies. In maize, infestations can reduce yields by up to 73%^20^. In its native range, the average economic losses were US$60 million per year in the southeastern USA from 1975 to 1983^21^. In Brazil, a more recent study showed a 34% reduction in grain maize yields, translating to US$400 million in losses, and US$600 million in control costs, per year^22,23^. In the invaded range, the estimated maize crop damage averaged 32% in Ethiopia and 47.3% in Kenya based on a survey conducted in 2017^24^. Day et al.^25^ estimated that the average FAW-related annual maize production losses in 12 African countries range between 21% and 53%. Similarly, the total annual loss in maize harvest is estimated to be between $2.2 to $5.5 billion for the 10 major maize-producing African countries^26^, and $9.4 billion for the whole continent^27^. Most reported yield losses likely reflect the composite impact of both permanent and transient FAW populations, as transient FAW populations can also cause substantial damage^28^.

**Fig. 1:**
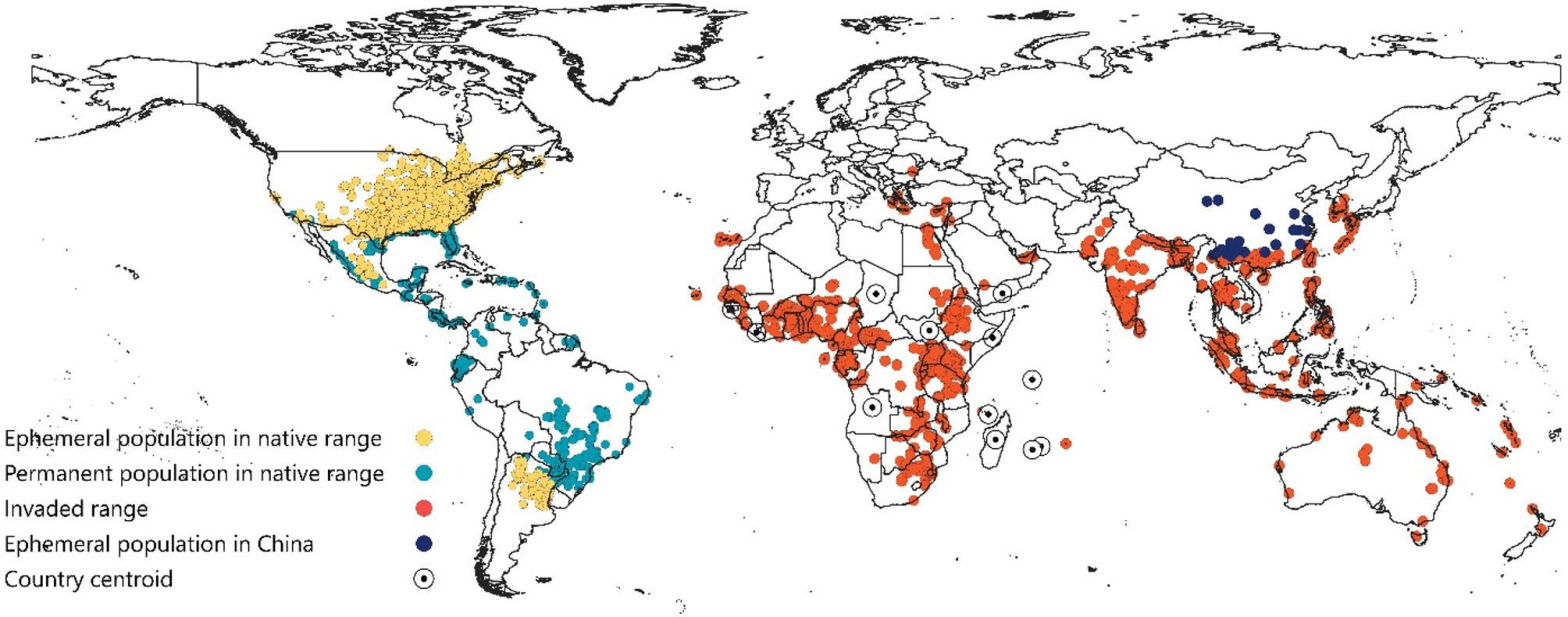
Confirmed presence records of *Spodoptera frugiperda* around the globe. Yellow occurrence records represent seasonal FAW populations within the native range. Light blue occurrence records show established FAW populations within its native range. Red occurrence records depict FAW populations within its invasive range. Dark blue occurrence records show the transient FAW populations based on population limits presented by Huang et al.^65^. Lastly, circled dots represent the centroid of a country/region (Angola, Yemen, Chad, Guinea-Bissau, Liberia, Madagascar, Mauritius, Mayotte, Réunion, Seychelles, Somalia, South Sudan), where FAW’s presence is confirmed only by a country centroid. This figure was created with QGIS version 3.36.2 (https://qgis.org/).

FAW’s established native range extends from northern Argentina to the southern USA^1,2^ (Fig. 1). Its year-round populations are confined to tropical and subtropical regions due to the absence of diapause mechanisms and its sensitivity to prolonged exposure to cold temperatures^29^. However, FAW adults migrate long distances during warmer months to seasonally suitable temperate regions where ephemeral populations can complete one or more generations. Johnson^7^ reported that FAW adults can fly 100 km overnight, while Rose et al.^30^ suggested they can migrate up to 1 600 km within 30 h under favourable conditions. Recent studies support these findings, indicating that FAW is capable of self-powered flights covering distances up to 117 km^31^, and can disperse over 1 100 km from its source location according to simulated migration trajectories in China^32^. In addition to natural dispersal, FAW larvae and pupae can be inadvertently transported as contaminants of traded commodities or as stowaways on airplane and vehicle vectors^12,33^ (Fig. S1).

FAW was added to the European and Mediterranean Plant Protection Organization (EPPO) A1 List of pests recommended for regulation as a quarantine pest in 2016. The EPPO A1 list includes pests not present in EPPO member countries that are regulated to prevent their entry. FAW was listed in Part A of Annex II to Commission Implementing Regulation (EU) 2019/2072^34^, as a pest not known to occur in the Union territory. However, it was transferred into the EPPO A2 List of pests recommended for regulation as a quarantine pest, due to its recent detection within some EPPO member countries^12^. A (Union) quarantine pest shall not be introduced into, moved within, held, multiplied, or released in the Union territory. Notably, FAW is among the top 10 most important quarantine pests for the EU that are recommended to be classified as “priority pests”^35^. A priority pest is expected to cause the most severe economic, environmental, and social impacts, compared to other Union quarantine pests. Consequently, FAW is established as a priority pest under the Commission Delegated Regulation (EU) 2019/1702^36^.

In Europe, FAW was first reported in Madeira (Portugal) in September 2023^37^. A month later, FAW adults were detected on the Greek mainland, in Laconia and Eastern Attica, followed by detections in Evvoia and eastern Crete^38^. Then, FAW was found in southern Romania^39^, and more recently, Malta announced the possibility of an FAW incursion on the island^40^. These developments signify the beginning of the FAW invasion into Europe, given that the climatic conditions in Greece and Malta may support the establishment of permanent FAW populations^41^.

Despite its presence in Europe, FAW continues to be regulated under Commission Implementing Regulation (EU) 2023/1143^42^, which formalizes measures to prevent its introduction, establishment, and spread within the Union territory. These measures involve annual surveys, the establishment of a contingency plan, and demarcated areas consisting of an infested zone and a 5–100 km wide buffer zone, where the pest is present, and eradication actions should be performed. Further phytosanitary measures target the importation of specific plant products into the Union, including peppers, eggplants, plants of *Chrysanthemum L.*, and maize. Such host plants may be imported into the Union if they originate from a country where FAW is absent, undergo official inspection, and be accompanied by a phytosanitary certificate. Despite these measures, the likelihood of FAW’s re-entry into the EU by natural dispersal remains high as it is already established in northern Africa and the Middle-East, well within its dispersal range. Mitigating the risk of re-entry via this pathway could only be achieved by controlling the pest at its source location (North Africa)^43^.

Bioclimatic models are valuable tools to estimate the climatically suitable areas for pest establishment^41,44–47^. In the case of FAW, various models have been applied, such as the process-based semi-mechanistic CLIMEX model^41,44–47^, the correlative Maximum Entropy (MaxEnt) model^48–50^, and ensemble modeling techniques^51,52^. However, there is no consensus regarding the projected potential distribution of FAW in Europe. For example, Ramirez-Cabral et al.^47^ used the CLIMEX model without differentiating between permanent and transient FAW populations in its native range. Consequently, this model estimated that most EU Member States (MSs) could support permanent FAW populations. Similarly, studies employing the MaxEnt model identified large parts of the European continent as climatically suitable for FAW establishment^48,50^. In contrast, other CLIMEX outputs, such as those from du Plessis et al.,^41^ Timilsena et al.,^44^ Senay et al.,^46^ and Wang et al.^45^ treat the native range data appropriately, and their resulting maps in Europe restrict suitable areas for establishment predominantly to the Mediterranean coast.

In addition to understanding FAW’s potential distribution, exploring the migration extent of its transient populations is crucial for assessing potential risk in Europe. While climatically suitable pockets for establishment are located along the Mediterranean coast, several studies suggest that much of the continent is suitable for transient FAW populations. However, limited information is available on FAW’s capacity to migrate to these seasonally suitable regions in Europe, with focus instead given to natural migration pathways from northern Africa into Europe^43,45^. Gilioli et al.^53^ indicates a heightened risk associated with transient FAW populations that could damage crop production up to 45^°^N. Yet, quantitative economic assessments of such damage remain sparse. CLIMEX models are useful for identifying at-risk areas and guiding subsequent analyses on FAW migration and its direct economic impact on key crops, such as grain maize and wheat^54–56^.

This study investigates the areas at risk of FAW invasion in Europe by assessing FAW’s potential distribution, migration capacity, and the potential direct economic impact on European grain maize production under a post-invasion “no-control” scenario. Firstly, we revised the projected areas suitable for permanent and transient distribution of FAW by using the CLIMEX model with updated parameter values on FAW’s climate-related developmental requirements, an expanded occurrence record dataset, and more recent climatic data, which have not been used in previous CLIMEX studies. Secondly, we examined the potential extent of seasonal FAW migration in Europe in relation to the areas modelled as suitable for transient populations by the CLIMEX model, by considering the migration patterns within FAW’s native range. Thirdly, given the modelled potential distribution and seasonal migration analysis, we quantified the potential direct economic impact of FAW invasion on European grain maize production, considering 13 MSs by using a partial budgeting approach^57^. To our knowledge, this is the first study to assess the potential economic damage that FAW can cause in Europe by integrating information on the pest’s potential distribution, migration capacity, and economic impacts. The findings aim to inform biosecurity responses as the continent faces the pest’s further establishment and spread.

## Methods

### Data collection and cleaning

#### Fall armyworm occurrence records

FAW occurrence records were obtained from the following sources: the Global Biodiversity Information Facility^58^ (GBIF), Butterflies and Moths of North America^59^ (BAMONA), the European and Mediterranean Plant Protection Organization^12^ (EPPO), and the Center for Agriculture and Biosciences International^60^ (CABI), as well as published datasets^44,52^ and occurrence records in the literature. For the occurrence records in the literature, we used the keywords “*Spodoptera frugiperda* [country name]” in Google Scholar for each country where FAW’s presence is confirmed by EPPO (last update for all sources: February 2024). All records reported in GBIF were acquired using the “rgbif”^61^ R package (R software version 4.3.3^62^) and selecting the corresponding GBIF taxon ID (5109855) for *Spodoptera frugiperda*.

We conducted a data cleaning process for the GBIF occurrence dataset by using the “CoordinateCleaner”^63^ (version 3.0.1) and “dplyr”^64^ R packages. We removed records with missing coordinates and those located on the null island (0,0). Records classified as “Absent”, “Fossil”, or “Living” specimens were also excluded. We also removed country centroids, country capitals, and those dated before 1950. Additionally, records with known inaccurate default values, high uncertainty, and those located at zoo and herbaria locations. Finally, records found in the ocean were removed from the dataset, and records without coordinates but with suitable location descriptions were geo-referenced. We then compiled all the occurrence records from the different sources in a single dataset and eliminated duplicates, resulting in 6 851 unique occurrence records. The occurrence records used in this study are depicted in Fig. 1, and the complete dataset is provided as Supplementary Information, including the source for each entry.

#### Climate data

We used the ‘CliMond CM_TC10: World’ climatology (C. Duffy, *unpublished data*) to fit the current climatic suitability of FAW under a rainfed and irrigation scenario. This dataset is interpolated in a 10-arc-minute gridded spatial resolution and consists of 30-year averages centred on 1995 (1981–2010) for daily minimum and maximum temperature (°C), monthly precipitation (mm), and relative humidity (%) recorded at 09:00 and 15:00 h. Additionally, we compared the CLIMEX output using the 1995-centered climate dataset with an equivalent one centred on 1975 (1961–1990), which is commonly used in CLIMEX studies (Figs. S2 – S3). Given climate change, an updated climate dataset representing the current climatic conditions could mean increased accuracy of the model outcomes.

### Modelling package and software

#### The CLIMEX model

CLIMEX (Hearne Scientific Software, Melbourne^66,67^ is a process-based climatic niche model that allows the estimation of the potential distribution of species as a response to the current or future climate. It incorporates parameters pertaining to how a species’ development is affected by climatic conditions, offering a comprehensive understanding of the pest’s ecological niche. Several suitability indicators are calculated for each pixel/unit area by incorporating occurrence data and information on climatic parameters and species-specific ecophysiological growth parameters. The model is based on the assumption that a species’ population experiences one or more (un)favourable periods for growth in a given year^67,66^. During the favourable season in a given location, the weekly temperature and soil moisture requirements for population growth are met, as described by the annual Growth Index (GI_A_). In contrast, an unfavourable season is characterized by population decline and no growth and can be characterized using a selection of up to four stress indices (cold, hot, dry, wet) and four stress interaction indices (cold-dry, hot-dry, cold-wet, and hot-wet). Integrating the GI_A_ and stress indices provides a single annual index of climatic suitability for a given location, the Ecoclimatic Index (EI). Both GI_A_ and EI range from 0 to the theoretical maximum of 100. An EI value of 0 at a given location indicates that the species cannot persist year-round. In this study, we set a climatic suitability classification system for both EI and GI_A_, as follows: unsuitable for EI = 0, marginal for 0 < EI ≤ 5, moderate for 5 < EI ≤ 15, suitable for 15 < EI ≤ 30, and optimal for EI > 30. We used the same classification system for every simulation, including the published CLIMEX models on FAW, to make the outputs comparable (Figs. S4, S5). QGIS (version 3.36.2) was used to project the CLIMEX output(s) and to visualize the different EI and GI_A_ classes for FAW.

**Table 1:** CLIMEX parameter values used for modelling the Climatic suitability of *Spodoptera frugiperda*, based on five published studies and the current study.

Parameters	Description	Unit	Ramirez-Cabral et al. 2017	du Plessis et al. 2018	Timilsena et al. 2022	Senay et al. 2022	Wang et al. 2023	Current study
Temperature index (TI)								
DV0	Lower temperature threshold	°C	12	12	12	8.7	12	9.4
DV1	Lower optimal temperature for growth	°C	22	25	25	24.6	25	26
DV2	Upper optimal temperature for growth	°C	27	30	30	32	30	30
DV3	Upper temperature threshold	°C	34	39	36	39.5	36	39.5
Moisture index (MI)								
SM0	Lower soil moisture threshold		0.1	0.15	0.15	0.15	0.15	0.1
SM1	Lower optimal soil moisture		0.7	0.8	0.8	0.8	0.8	0.65
SM2	Upper optimal soil moisture		0.9	1.5	1.5	1.5	1.5	1.5
SM3	Upper soil moisture threshold		1.5	2.5	2	2.5	2.5	2
Cold stress (CS)								
TTCS	Cold stress temperature threshold	°C	8	12	8	8.7	8	9.4
THCS	Cold stress accumulation rate	week^− 1^	−0.00001	−0.001	−0.005	−0.001	−0.005	−0.003
Heat stress (HS)								
TTHS	Heat stress temperature threshold	°C	38	39	39	39.5	39	39.5
THHS	Heat stress accumulation rate	week^− 1^	0.001	0.005	0.0025	0.005	0.0025	0.005
Dry stress (DS)								
SMDS	Soil moisture dry stress threshold		0.1	0.1	0.1	0.1	0.1	0.1
HDS	Dry stress accumulation rate	week^− 1^	−0.001	−0.005	−0.005	−0.005	−0.005	−0.005
Wet stress (WS)								
SMWS	Soil moisture wet stress threshold		1.5	2.5	2	2.5	2.5	2
HWS	Wet stress accumulation rate	week^− 1^	0.001	0.002	0.01	0.002	0.001	0.01
Limiting conditions								
PDD	Length of growing season / Minimum degree day sum needed to complete a generation	°C days	559	600	400	559	391.61	392
Other								
Irrigation		mm day^− 1^	–	2.5	2.5	–	2.5	2.5

#### Model fitting

The climatic suitability of FAW was projected by using the “Compare Location (one species)” module in CLIMEX (version 4.1.1.0)^67^. We chose the set of CLIMEX parameter values for FAW of the most recently published model^45^ as a starting point. These parameter values were revised by considering (i) recent literature not used in previous CLIMEX models on climatic requirements for the growth and development of FAW, (ii) additional FAW global occurrence records, and (iii) other published CLIMEX models on FAW^41,44–47^. The model fitting was an iterative process. The main goal was to fit the model to the occurrence records representing permanent FAW populations, in regions where the limits of such populations are known, in the area with positive EI (EI > 0). We then confirmed that the occurrence points representing transient populations were within areas where the model indicates positive GI_A_ (GI_A_>0) and EI = 0. FAW transient points in Fig. 1 are based on known permanent-transient population boundaries in the Americas and Canada^68^, and by Huang et al. in China^65^. It was not possible to accurately set permanent FAW population limits in southern Africa, Australia, and New Zealand due to insufficient data. In addition to fitting the distribution data, parameter values were also required to be biologically plausible. We compare the parameter values of the current study with those reported in five published CLIMEX models on FAW in Table 1. Further details and justification for the parameter values used in this study are discussed below.

#### Growth indices

##### Temperature index (TI)

Senay et al.^46^ chose a lower temperature threshold for development (DV0) of 8.7 °C as the minimum threshold for development averaged across all life stages of FAW, a result based on a second-degree polynomial regression^69^. However, because pupation is required to reach the sexually mature adult life stage and complete a generation, we used 9.4 °C as DV0, corresponding to the minimum temperature threshold for FAW pupal survival (same study^69^. The chosen DV0 value is similar to the field findings by Yang et al.^70^, who showed that FAW pupae can overwinter in the northern limit of Kunming in January 2020, where the average monthly temperature was 9.24 °C. Following the parameter values selected by du Plessis et al.^71^, the temperature range for optimal development was set from 26 °C (lower threshold—DV1) to 30 °C (upper threshold—DV2). and supported by more recent data on temperature-dependent development and survival rates^72,73^. According to Valdez-Torres et al.^69^, the maximum temperature threshold for FAW is 39.8 °C, thus the upper temperature for development (DV3) was rounded to 39.5 °C.

##### Moisture index (MI)

Recent experiments indicated that approximately 30% of FAW larvae burrowing in soil with 0% soil moisture were able to pupate successfully^74^. However, host plants cannot tolerate a complete absence of soil moisture, so we set the lower soil moisture threshold (SM0) at 0.1. The lower optimal soil moisture (SM1) was adjusted to 0.65, as FAW is highly polyphagous, and many plant species grow well at low moisture levels. The upper optimal soil moisture (SM2) and the upper soil moisture threshold (SM3) remained unchanged from the majority of previous CLIMEX models at 1.5 ^41,45,46^ and 2^44^, respectively. This allowed growth in the wettest areas of FAW’s distribution.

#### Stress indices

##### Cold stress (CS)

 FAW potential distribution appears sensitive to the cold stress parameters. The cold stress temperature threshold (TTCS) was set to 9.4 °C, in line with recent data supporting that FAW survival at different development stages decreases significantly when exposed to temperatures below 9 ± 0.5°C^75–77^. The cold stress accumulation rate (THCS) was adjusted to -0.003 week^− 1^ to allow FAW development in the cooler limits of its permanent populations^2^. Specifically, this included records along the Nile River Basin in Egypt, the Mediterranean coast of northern Africa, close to the border of Niger and Nigeria, north Argentina, the Australian state of Queensland, and southern China.

##### Heat stress (HS)

The heat stress temperature threshold (TTHS) was set to 39.5 °C, which is the value for DV3. The heat stress accumulation rate (THHS) was set to a moderate level of 0.005 week^− 1^ following du Plessis et al.^41^ and Senay et al.^46^. This allowed persistence in the hottest areas of FAW’s range.

##### Dry stress (DS)

The soil moisture dry stress threshold (SMDS) was set to 0.1, which is the value for SM0. The dry stress accumulation rate (HDS) was then adjusted to -0.005 week^− 1^ to limit the suitability projections to the tropical and subtropical areas where permanent FAW populations occur. This is also in agreement with most published CLIMEX models on FAW^41,44–46^.

##### Wet stress (WS)

The soil moisture wet stress threshold (SMWS) was set to 2 to be consistent with the value of SM3. Then, in accordance with Timilsena et al.^44^, the wet stress accumulation rate (HWS) was set to 0.01 week^− 1^ to restrain FAW suitability to the wetter tropical and subtropical regions where its permanent populations occur.

#### Minimum degree day sum (PDD)

This parameter describes the minimum required number of growing degree days above DV0 to complete a generation. Based on du Plessis^71^ estimates, FAW needs 391.61 °C days for egg-to-adult development. We rounded this parameter value to the nearest whole number (392 °C days) since CLIMEX is insensitive to such precision.

#### Irrigation

To account for irrigation, we ran the CLIMEX model with an irrigation scenario applied as an additional 2.5 mm day^− 1^ as a top-up above the default rainfed scenario, throughout the year. The Global Map of Irrigated Areas (GMIA) is used to define where the irrigation scenario shall be applied^78^. This assumes that no irrigation was added in areas where the rainfall is already equal to or greater than 2.5 mm day^− 1^, under the rainfed scenario. In the case of FAW, dry areas in North Africa, such as the Nile River, and in Pakistan and Yemen, where FAW occurs permanently and irrigation is applied, do not appear as climatically suitable under rainfed conditions^44^, supporting the hypothesis that they are able to persist in these locations only due to the presence of irrigation.

### Modelled migration distances in Europe

FAW regularly migrates long distances during spring and summer. In North America, transient occurrence records have demonstrated the pest’s dispersal throughout the USA and southern Canada, causing substantial seasonal damage^12,68^. The spatial pattern of this seasonal dispersal was estimated using a subset of the original distribution dataset (n’=1 831) that consists of the transient occurrence records in the USA and Canada. These records are characterized by EI = 0 and GI_A_>0 in the afore-described CLIMEX model. Thus, we assessed the minimum distance of each transient occurrence point from the nearest area (hub) suitable for year-round population persistence (EI > 0; southern coast of the USA). Weinberg et al.^79^ employed a similar method to estimate the spatial pattern of seasonal dispersal of the southern armyworm (*Spodoptera eridania*). The QGIS “Distance to nearest hub (line to hub)” algorithm was used to compute the distance between each transient occurrence record and the closest destination layer (EI > 0 zone).

To reduce overestimation bias in our analysis, we excluded 115 occurrence records from the initial data subset (*n* = 1 946) because they were located on Bermuda Island (EI > 0). Including these records would imply that FAW flew more than 1 000 km overseas in a single event. The resulting dataset provided the distribution of minimum distances between transient FAW records and the nearest permanent establishment hub (EI > 0). Based on this distribution, we created buffer zones around the modelled EI > 0 area (hub)^79^. These buffer zones represent dispersal frequency zones and illustrate areas that may be accessible to FAW transient populations, with the risk of migration diminishing as the distance from the EI > 0 area increases (Fig. S6). For instance, Fig. 3 shows two buffer zones derived from the 50th and 100th percentile of the FAW migration distances distribution, indicating median and maximum potential extents of FAW natural migration, at least under average climatic conditions. A GI_A_>0 area that falls adjacent to, or within these buffer zones, indicates potential crop exposure to migrating populations. Conversely, GI_A_>0 areas outside the buffer zone of maximum distance are assumed to be inaccessible to FAW by flight.

Assuming that FAW observed migratory capacity in Europe is broadly similar to its behaviour in its native range, we applied these dispersal frequency zones across the European continent. The results were considered alongside our CLIMEX model outcomes, which identify climatically suitable areas for seasonal FAW populations (GI_A_>0) within Europe.

### Direct economic impact

#### Economic data

National average values of grain maize revenues and operating costs from 2010 to 2020 were obtained from the EU Cereal Farms database of the Farm Accountancy Data Network (FADN) for 13 EU MSs: Austria, Bulgaria, Croatia, France, Germany, Greece, Hungary, Italy, Poland, Portugal, Slovakia, Slovenia, and Spain. We also used EUROSTAT to obtain information on the grain maize cultivated area for each MS. These MSs contributed more than 80% of the total grain maize production in the EU-27 for the years 2022 and 2023, respectively (own calculation based on EUROSTAT). Moreover, the European Food Safety Authority (EFSA) provides expert-elicited estimates on grain maize yield losses due to FAW, based on formal EKE methodology^80–82^. In the case of grain maize, losses are the consequences of plant decline and rejected, and unharvested cobs. A key assumption of the EKE data is that similar levels of yield loss occur in both permanent and transient FAW population areas.

#### Direct economic impact

To estimate the direct economic impact of FAW invasion on European grain maize production, we employed a partial budgeting method. Partial budgeting is an appropriate method to evaluate the economic consequences of a shock, such as a pest invasion, by accounting for the potential economic benefits and losses^57^. In the case of FAW, direct impacts include solely negative effects, namely yield losses and additional operating costs. The direct economic impact was estimated over the study area where the CLIMEX model projected the establishment of FAW (Mediterranean coast) but was limited to where the migratory behaviour and capacity analysis suggested the likelihood of seasonal populations. However, this encapsulated all of the 13 EU MSs included in the FADN dataset. We performed the analysis under the irrigation scenario since it better reflects FAW’s ecological niche. Furthermore, the baseline grain maize gross margins were computed and compared with the gross margins with FAW presence.

The direct economic impact assessment was conducted under specific assumptions. Firstly, we performed the analysis under a post-invasion no-control scenario^83^, assuming that no additional regulatory or control measures are in place after the invasion, and thus no change in the operating costs. This “worst-case” scenario provides a benchmark for the potential scale of damages due to FAW without intervention. Secondly, we also assumed a complete occupancy of the climatically suitable area (EI > 0) in Europe (Mediterranean coast) and that the northward migration starts over from that area each year. This assumption aligns with the pest’s observed behaviour in its native (and invaded) range, where areas with permanent FAW populations serve as sources for seasonal dispersal. Thirdly, the migratory capacity of FAW follows a similar pattern as in the USA and Canada, thus, the representation of accessible zones outside the area of permanent establishment remains consistent in Europe. Fourthly, the probability of attack is inversely related to the distance from the permanent FAW establishment area, reflecting the diminishing annual likelihood of successful migration as distance increases. Therefore, northern EU MSs bear a smaller risk than those further south. Finally, the economic model only accounts for the natural migration of the pest through flight; other dispersal pathways, such as trade, were not considered in the analysis.

#### Baseline gross margins

We used the gross margin per hectare (€/ha) as a baseline representing the current economic state of EU grain maize in each MS. Gross margins capture the difference between revenue and operating costs, providing a more accurate reflection of farm-level profitability than just revenues alone. The gross margin is calculated as follows:

1 $$GM_{i}^{{baseline}} = \frac{1}{n}\sum {\:_{{t = 1}}^{n} } (R_{{i,t}} - OC_{{i,t}} )$$

$\:{GM}_{i}^{baseline}$ is the average grain maize gross margin per hectare (€/ha) which is determined by the difference between annual revenues per hectare $\:{R}_{i,t}$ and operating costs per hectare $\:{OC}_{i,t}$ in each MS $\:i$ and for each year $\:t$ over the period 2010–2020. The $\:{OC}_{i,t}$ component takes into account several cost categories, such as specific costs (€/ha) (seeds, fertilizers, crop protection, water, other specific costs) and non-specific costs (€/ha) (motor fuels and lubricants, machines, buildings, contract work, energy (electricity, heating fuels) and other direct costs).

#### Gross margins with FAW

The gross margins under FAW presence represent the post-invasion grain maize gross margins were calculated as:

2 $$\begin{array}{*{20}c} {GM_{i}^{{FAW}} = \bar{R}_{i} \left( {1 - PP_{i} \frac{{YL_{{i,s}} }}{{100}}} \right) - \overline{{OC}} _{i} } \\ \end{array}$$

$\:{GM}_{i}^{FAW}$is the grain maize gross margin with FAW presence in MS $\:i$ (€/ha), $\:{\stackrel{-}{R}}_{i}$ and $\:{\stackrel{-}{OC}}_{i}$ are the average revenue and operating costs (€/ha) in MS $\:i$, respectively, $\:{YL}_{i,s}$ is the EKE yield loss for MS $\:i$ and yield loss scenario $\:s$, and $\:{PP}_{i}$ is the probability of FAW presence for MS $\:i$.

The $\:{PP}_{i}$ parameter accounts for each MS’s annual risk of invasion based on its geographical proximity to areas of FAW establishment (EI > 0). We assume that northern countries face lower risks compared to those located in the south, which are closer to the area of permanent establishment. More specifically, $\:{PP}_{i}$ captures the probability of FAW presence in a MS, considering the pest’s migratory capacity (flight distance), whereas $\:{YL}_{i,s}$ incorporates the expert-elicited yield losses^82^ that may be affected by several factors, including FAW population abundance and short generation time (Table S1).

To estimate the $\:{PP}_{i}$ for each MS $\:i$, we used an empirical cumulative distribution function (ECDF). We utilized the data subset that includes the distance distribution of transient FAW occurrence records in North America. An ECDF value represents the proportion of all observed migration distances that are less than or equal to a specific distance away from the projected area of permanent FAW establishment. Thus, ECDF allows us to assign a probability to a specific distance of interest from the EI > 0. The $\:{PP}_{i}$ parameter is calculated as:

3 $$\begin{array}{*{20}c} {PP_{i} = 1 - F\left( {D_{i} } \right)} \\ \end{array}$$

where $\:{D}_{i}$ denotes the distance from the centroid of MS $\:i$ to the nearest area with EI > 0 along the Mediterranean coast and $\:F\left({D}_{i}\right)\:\in\:\left[\text{0,1}\right]$ is the ECDF value at that distance. This approach assumes the inverse relation between distance and invasion risk with proximity to EI > 0 corresponding to higher risks of FAW invasion (Figs. S7, S8).

To extend the per-hectare direct impacts (€/ha) to national levels, we scaled the per-hectare gross margins by the total cultivated area of grain maize in each MS. In particular, we multiplied the gross margins (€/ha) by the total grain maize cultivation area, based on EUROSTAT data from 2013 to 2023, for each MS.

Lastly, the direct economic impact $\:{DEI}_{i,s}$ is calculated by comparing the baseline gross margins to those under FAW presence:

4 $$\begin{array}{*{20}c} {DEI_{{i,s}} = GM_{i}^{{baseline}} - GM_{{i,s}}^{{FAW}} } \\ \end{array}$$

Three scenarios were considered to capture the bandwidth of the potential impact of FAW on European grain maize production: a best-case, moderate-case, and worst-case scenario that assume a yield loss percentage equal to the 2.25th, 50th, and 97.5th percentiles of the MS-specific yield loss distribution, respectively. These yield loss scenarios are based on the EFSA EKE data^80–82^, which incorporate various assumptions, including the effectiveness of current, untargeted control measures against FAW, climate conditions, and damage type. One example of such indirect management is the chemical control against *Helicoverpa armigera*, which may also have a collateral benefit tackling FAW populations^84^. Table S1 provides the reasoning behind each scenario, and Table S2 includes the exact values.

## Results

### Potential distribution of *Spodoptera frugiperda* under current climatic conditions

**Fig. 2:**
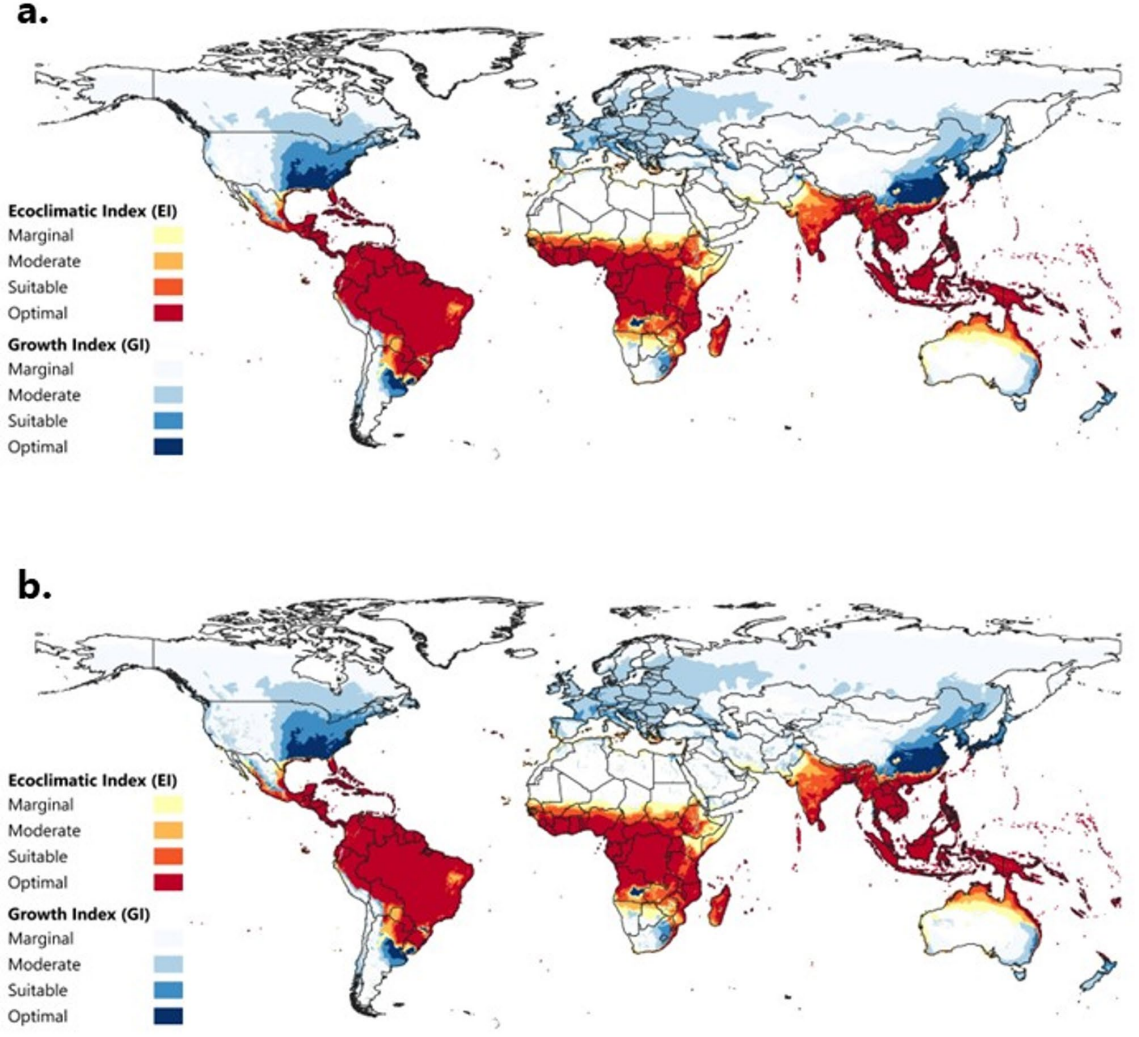
Global climatic suitability of *Spodoptera frugiperda* modeled using the Compare Locations module in CLIMEX v4.1.1.0 ran with 30-year average climatic data centred on 1995 (CM_TC10_1995_v1) (**a**) under rainfed conditions and (**b**) under a composite irrigation scenario (2.5 mm day^-1^ applied as top-up). The Ecoclimatic Index (EI) and Growth Index (GI_A_) are the outputs of the parameters used in Table 1. The EI gradient (yellow-red) represents areas suitable for all year-round FAW population establishment. The GI_A_ gradient (light blue-dark blue) depicts areas suitable for seasonal population growth and migration. The figure was created with QGIS version 3.36.2 (https://qgis.org/).

The modelled potential FAW global climatic suitability accords with the pest’s known present distribution and population limits (Fig. 2), also considering its migratory nature. Following the Köppen-Geiger climate classification system^85^, FAW is established in tropical, subtropical, and Mediterranean climates. More specifically, areas with tropical climates (tropical rainforest, tropical monsoon, and tropical savanna), as well as subtropical climates (humid subtropical, and subtropical highland) exhibit the highest Ecoclimatic Index values (EI > 30), indicating optimal climate conditions for FAW. Notably, irrigation plays a substantial role in expanding the climatic suitability in drier climates, enabling permanent FAW establishment in pockets of hot semi-arid climates, and hot-summer Mediterranean climates. The model also indicates that areas with humid continental, Mediterranean (hot-summer, cold-summer, warm-summer), and all humid subtropical climates are suitable for transient FAW populations, exhibiting moderate Growth Index (GI_A_) values (GI_A_ >15, EI = 0). Areas with desert climates (hot, cold, cold semi-arid) appear marginally to moderately suitable for permanent populations, but only if irrigation is applied.

In Europe, the CLIMEX model projects the Mediterranean coast as a critical area for FAW establishment under both rainfed and irrigation scenarios (Figs. 2 and 4). In particular, southern portions of Portugal, Spain, Greece, Italy, the Balearic Islands, Cyprus, and Turkey exhibit favourable conditions for FAW populations to persist. The model further identifies pockets of suitable conditions in the Mediterranean parts of northern Egypt, Tunisia, Libya, Morocco, and Algeria. Furthermore, our analysis suggests a substantial proportion of Europe is vulnerable to seasonal migratory FAW incursions. Areas exhibiting moderate to optimal GI_A_ values expand far beyond the area modelled as suitable for year-round population persistence. Under natural rainfall conditions, higher GI_A_ values are limited to areas in the southwestern and eastern parts of France, northern Italy, and the western Balkans, whereas the vast majority of Europe was modelled as marginal to moderately suitability. Under the irrigation scenario, MSs located in the south, west, and east of Europe are highly suitable for migrating FAW populations (GI_A_>15). FAW presence was detected on mainland Greece (Laconia and Eastern Attica) in areas modelled as being suitable for population persistence, unlike the occurrence record reported in Romania, which appears only seasonally suitable for population growth. Additional information on FAW’s potential distribution based on our CLIMEX model is included in the Supplementary Information.

### Extent of seasonal dispersal – migration capacity

**Fig. 3:**
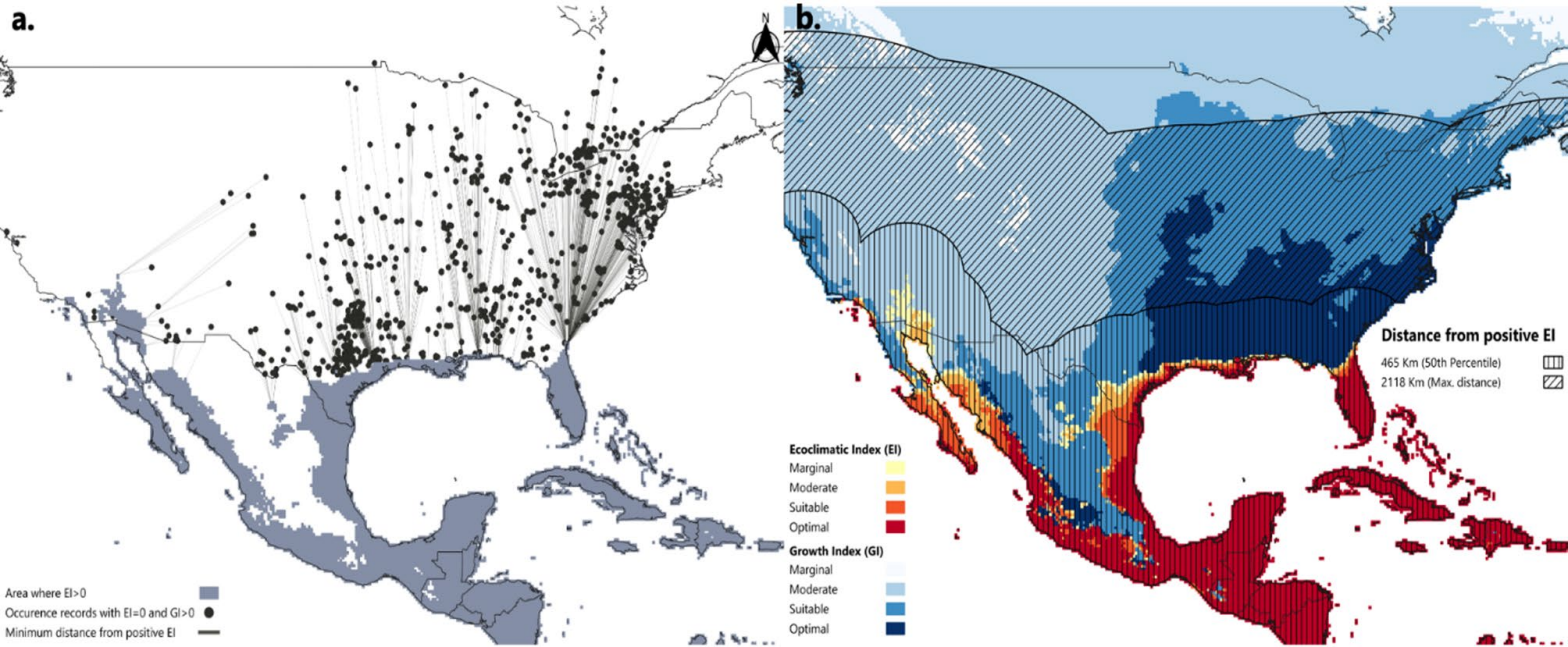
(**a**) Occurrence records of *Spodoptera frugiperda* representing ephemeral populations in the USA and Canada. These occurrence records were used for the “distance to nearest hub (line to hub)” analysis. (**b**) Projected climatic suitability modeled using the Compare Locations module in CLIMEX v4.1.1.0 ran with 30-year average climatic data centred on 1995 (CM_TC10_1995_v1). FAW dispersal frequency zones are depicted using cross-hatching buffer zones. The vertical hatching buffer zone extends to a 465 km distance from the area of permanent establishment (50th percentile) and the diagonal hatching buffer zone to a 2118 km distance (maximum recorded distance from EI > 0). The figure was created with QGIS version 3.36.2 (https://qgis.org/).

Based on the observed migration dynamics in the USA, the “distance to the nearest hub (line to hub) analysis” suggests a wide range of migration (Fig. 3). If all of the FAW historical occurrences represent transient populations that migrated by flight, the resulting distribution appears to be multimodal and right-skewed, suggesting that there are several peaks, and most occurrences are concentrated at lower distances (closer to EI > 0 area). More specifically, the observed migration distance ranges from 30 km (2.5th percentile) to 1 624 km (97.5th percentile), with a median of 465 km away from the nearest area with a positive EI value. It is worth noting that FAW seasonal range expansion during warmer months may surpass 2 000 km, as the farthest occurrence record from the EI > 0 area is located in Ontario, Canada. The larger distances are doubtless the result of multiple migration steps spanning more than one generation. In any given year, FAW may not be present in each of the northern states in the USA, depending on stochastic factors such as the growing conditions in the mid-latitude states and the wind currents during the further northern migrations.

**Fig. 4:**
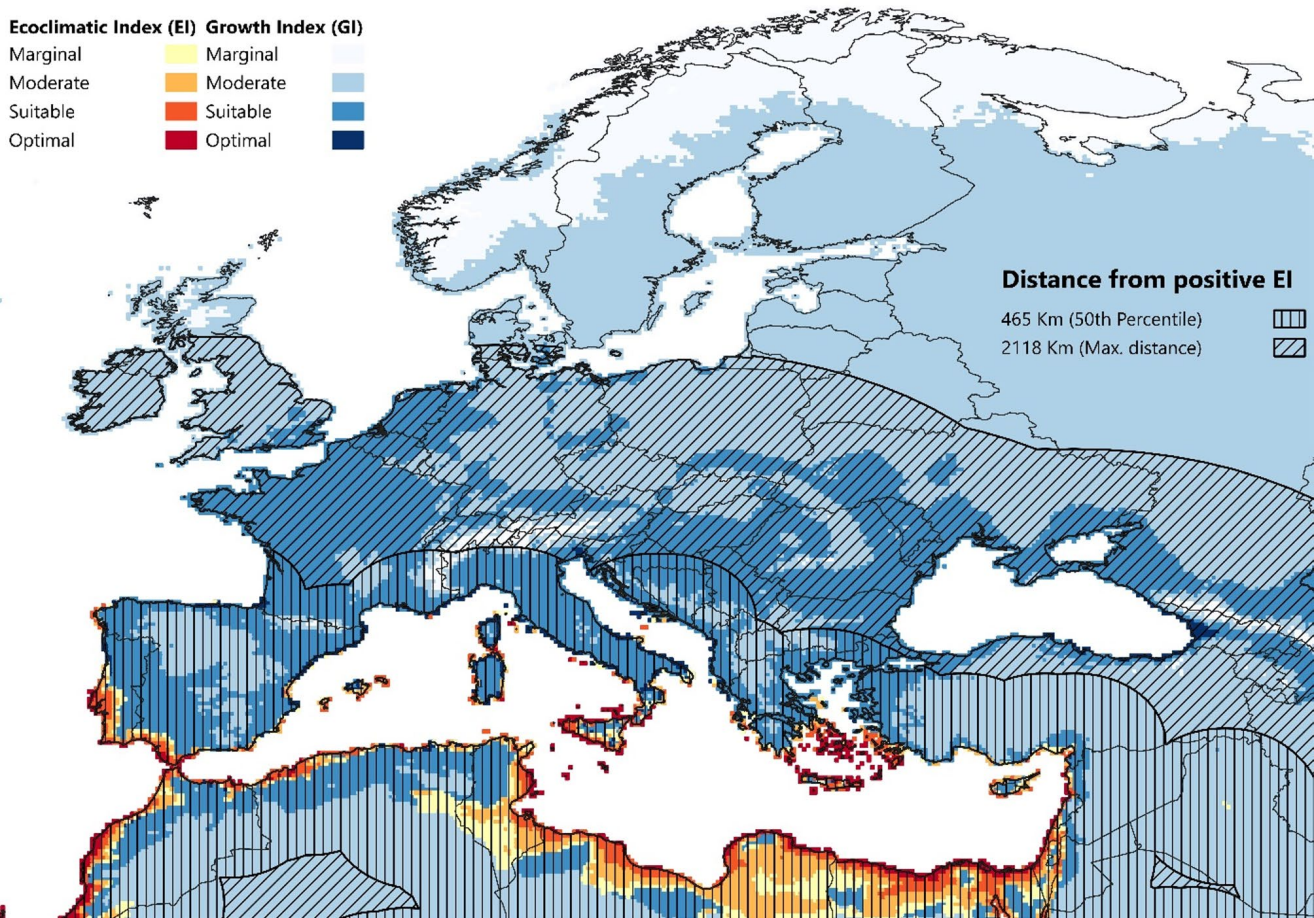
Projected climatic suitability of *Spodoptera frugiperda* in Europe modeled using the Compare Locations module in CLIMEX v4.1.1.0 ran with 30-year average climatic data centred on 1995 (CM_TC10_1995_v1). FAW dispersal frequency zones are depicted using cross-hatching buffer zones and are based on FAW’s migratory patterns in the USA and Canada. The vertical hatching buffer zone extends to a 465 km distance from the area of permanent establishment (50th percentile), and the diagonal hatching buffer zone to a 2118 km distance. The buffer zones were obtained, using the “distance from nearest hub” function in QGIS version 3.36.2 (https://qgis.org/).

Mediterranean European MSs bear the highest risk of hosting ephemeral FAW populations each year. Countries such as Portugal, Spain, Malta, Italy, Greece, southern France, and countries in the southwest Balkan Peninsula are located within 465 km (50th percentile) away from the nearest area with EI > 0 (Fig. 4). Furthermore, countries including Austria, Switzerland, Hungary, Serbia, and large portions of France, Germany, Czech Republic, Slovakia, and Romania are located within 1 079 km (75th percentile) away from the suitable habitat for permanent FAW establishment (Fig. S9). Finally, FAW’s annual migration may reach northern European countries, such as Poland, northern Germany, the Netherlands, Ireland, and the UK, excluding Scotland, when considering the furthest observed migration distance in FAW’s native range (= 2 118 km). Evidently, these areas are less likely to host seasonal FAW populations compared to those located within the 50th percentile of the distribution. All of these countries at risk from migratory populations are also modelled as being climatically suitable for transient FAW populations.

### Potential direct economic impact

**Fig. 5:**
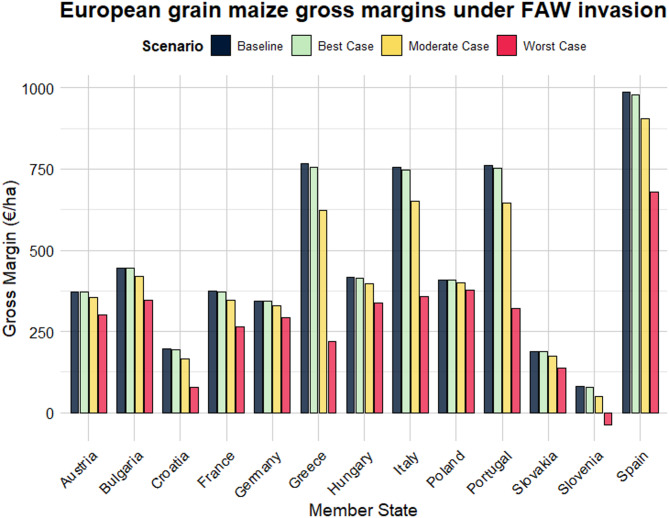
The potential annual direct economic impact of *Spodoptera frugiperda* on grain maize gross margins (€/ha) in different EU Member States. Dark bars represent the current (baseline) average gross margins (€/ha) with FAW absence. Green, yellow, and red bars represent the annual gross margin (€/ha) after FAW invasion, corresponding to the 2.25th, 50th, and 97.5th percentile of the Member State-specific yield loss distribution, respectively. The figure was created with the ggplot2 package in R Studio version 4.3.3. (https://www.r-project.org/)

Table 2 presents the average annual impact of FAW invasion on European grain maize, including the resulting gross margin (€/ha) and direct economic impact (€/ha) across different EU MSs. Baseline grain maize gross margins for grain maize (€/ha) varied considerably across the 13 MSs analysed (Fig. 5). Slovenia, Slovakia, and Croatia exhibit the lowest total baseline gross margins at 81 €/ha, 189 €/ha, and 196 €/ha, respectively. In contrast, MSs such as Spain, Greece, and Portugal have the highest baseline gross margins, at 985 €/ha, 766 €/ha, and 760 €/ha, respectively.

**Table 2:** Average annual grain maize gross margins and direct economic impact (€/ha) of *Spodoptera frugiperda* in Europe under different yield loss scenarios.

	Gross margin (€/ha)				Direct economic impact (€/ha)		
Member state	Baseline	Best case	Moderate case	Worst case	Best case	Moderate case	Worst case
Austria	373	372	354	300	1	19	73
Bulgaria	446	444	420	346	2	26	100
Croatia	196	194	166	80	2	30	116
France	375	373	346	264	2	29	111
Germany	343	342	330	293	1	13	51
Greece	766	755	623	220	11	143	546
Hungary	417	415	396	337	2	21	80
Italy	756	748	652	357	8	104	398
Poland	408	408	400	377	1	8	31
Portugal	760	752	645	321	9	115	440
Slovakia	189	188	175	137	1	14	52
Slovenia	81	78	50	−37	2	31	118
Spain	985	979	905	679	6	80	306
Average	469	465	420	283	4	49	186

Under the best-case scenario, we observe negligible differences in annual grain maize gross margin values for most MSs. In relative terms, gross margin losses (€/ha) range from 0.15% in Poland to 3% in Slovenia (Table S3). However, in absolute terms, Greece experiences the highest decrease, of 11 €/ha (Table 2, Fig. S10). Similarly, under the most likely (moderate case) scenario, the economic impact in terms of gross margin loss ranges from 2% in Poland to 62% in Slovenia. Greece remains the most impacted MS, with 143 €/ha losses in gross margin, followed by Portugal and Italy, with 115 €/ha and 104 €/ha losses, respectively. Lastly, under the worst-case yield loss scenario, Mediterranean MSs experience the highest economic impacts (€/ha). Gross margin losses rise to 546 €/ha in Greece, 440 €/ha in Portugal, 398 €/ha in Italy and 306 €/ha in Spain. Notably, based on our results, grain maize cultivation appears unprofitable in Slovenia, exhibiting negative gross margins (-37 €/ha).

**Fig. 6:**
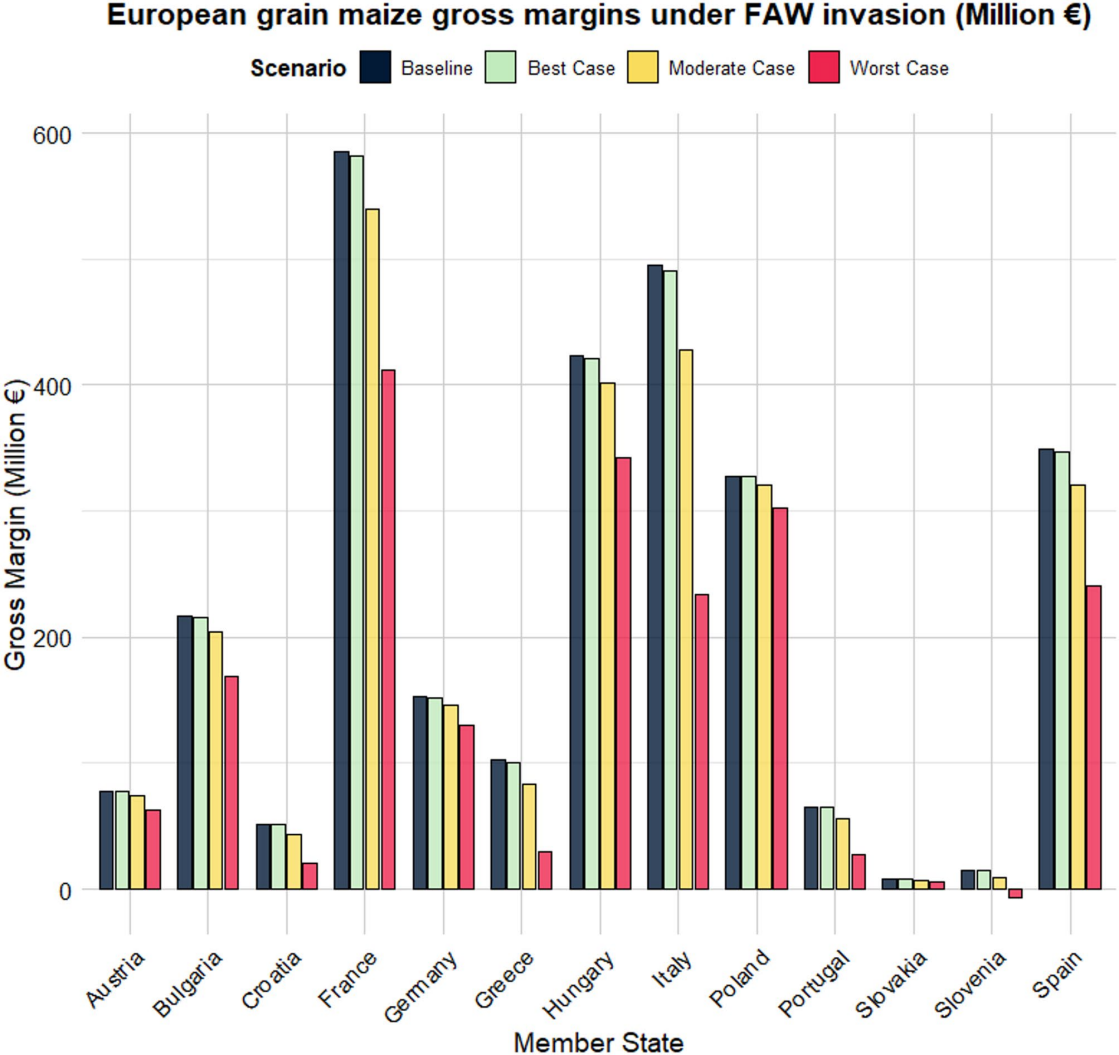
The potential annual direct economic impact of *Spodoptera frugiperda* on grain maize gross margins (in million €) in different EU Member States. Dark bars represent the current (baseline) average gross margins (€/ha) with FAW absence. Green, yellow, and red bars represent the annual gross margin (€/ha) after FAW invasion, corresponding to the 2.25th, 50th, and 97.5th percentile of the Member State-specific yield loss distribution, respectively. The figure was created with the ggplot2 package in R Studio version 4.3.3. (https://www.r-project.org/)

Table 3 provides the gross margins and direct economic impacts in million € at the MS level. Baseline annual gross margins range from 7 million € in Slovakia to 585 million € in France. Following France, Italy, Hungary, and Spain are the main grain maize-producing MSs, averaging 496 million €, 423 million €, and 349 million € per year, respectively (Fig. 6).

**Table 3:** Average annual gross margins and direct economic impact (in million €) of *Spodoptera frugiperda* on grain maize production in Europe under different yield loss scenarios.

	Gross margin (million €)				Direct economic impact (million €)		
Member state	Baseline	Best case	Moderate case	Worst case	Best case	Moderate case	Worst case
Austria	78	78	74	63	0.3	4.0	15.2
Bulgaria	217	216	204	168	1.0	12.8	48.7
Croatia	52	51	44	21	0.6	8.0	30.7
France	585	582	540	412	3.5	45.3	172.9
Germany	152	152	146	130	0.5	5.9	22.6
Greece	102	101	83	29	1.5	19.1	72.8
Hungary	423	421	402	342	1.6	21.2	81.1
Italy	496	490	427	234	5.3	68.4	261.3
Poland	328	327	321	303	0.5	6.6	25.0
Portugal	65	65	55	28	0.8	9.9	37.8
Slovakia	7	7	7	5	0.0	0.5	2.1
Slovenia	15	15	9	-7	0.4	5.8	22.2
Spain	349	347	321	241	2.2	28.4	108.6
Sum	2 870	2 852	2 634	1 969	18.1	235.9	901.1

Our findings indicate that annual gross margin losses range from as low as 0.04 million € in Slovakia to 5.3 million € in Italy, under the best-case scenario. Evidently, the results differ substantially between countries, especially when compared with those of gross margins per hectare. Under the moderate and worst-case scenarios, Slovakia remains the least impacted in terms of potential direct economic losses. In contrast, Italy, France, and Spain are at greatest risk (Fig. S11). Total direct economic losses of these MSs range from €28 million to €68 million per year, under the moderate case scenario, and from €109 million to €261 million per year, under the worst-case scenario. Summing the estimated losses across all the 13 MSs considered, we found annual direct economic losses of €18 million in the best-case scenario, €236 million in the most likely moderate-case scenario, and €901 million in the worst-case scenario.

## Discussion

Our study investigated the potential economic impact of the fall armyworm (FAW) on European grain maize gross margins production under a “no control” scenario, accounting for FAW’s potential distribution and migratory behaviour. Since crop damage in terms of yield losses is the primary FAW impact^2,28,60^, this study focused only on the annual direct economic impact. The research yielded three key findings. Firstly, FAW poses a considerable risk to Europe, as our CLIMEX model identified several areas along the Mediterranean coast that can support permanent population establishment. Secondly, if, as appears likely, FAW becomes established throughout the modelled climatically suitable zone in Europe, transient populations may spread naturally to most EU MSs during warmer months. Even failing this, established populations in northern Africa and the Middle East will likely provide source populations for seasonal migrations into Europe. Lastly, our results demonstrate that sizable economic impacts can be expected from FAW invasion and establishment in Europe with grain maize gross margin losses reaching up to 546 €/ha or €261 million per year in vulnerable areas.

Our CLIMEX model accurately reflects the dynamic range of FAW, clearly distinguishing between areas where the pest can sustain year-round populations and those where it can seasonally extend its geographical range during the favourable warmer seasons. The pattern also accurately captures the observed range dynamics in China. This attests to the accuracy of the model’s results when projected onto areas where FAW does not yet occur. The model suggests that FAW can establish permanent populations in the world’s tropical, sub-tropical, and some Mediterranean, climates. The tropical and sub-tropical origins of FAW have been well-recognized for nearly a century^10^. Further, more recent evidence indicates that Mediterranean climates are suitable as well^2,44,45,52,53^.

In Europe, regions such as the Balearic Islands, Malta, and considerable parts of southern Portugal, Greece, and Sicily are projected to be climatically suitable for FAW establishment. Additional suitable pockets extend along the coastal areas of Spain, Italy, Cyprus, and Turkey, as well as North African countries. Southern Europe faces a heightened risk of annual FAW reinvasions, especially if the pest fully colonizes suitable areas in North Africa and the Middle East^45^. Considering the pest’s natural or wind-mediated dispersal capability, no interventions are possible to prevent its seasonal reentry from northern Africa into Europe, beyond perhaps supporting management efforts in those (source) areas^43,68^. Similarly, assuming FAW occupies all suitable areas for establishment in Europe, targeted management efforts should focus on containment and control at source locations to mitigate the risk of northward migration and broader continental spread of transient populations Providing IPM tools including automated monitoring technology (e.g., detection technologies, efficient smart traps and area wide informatics and modelling platforms) and new environmentally friendly control technologies (e.g. new strains of BtK and entomopathogens) will be necessary for implementing the management strategies. Notably, recent detections of FAW in Greece^38,86^ underline the urgency for strengthening surveillance efforts across the Mediterranean, which would benefit from a better understanding of its potential impacts in Europe.

Our results accord with previous studies^41,44–46^ indicating that the Mediterranean coast hosts FAW overwintering pockets, further supporting its vulnerability to FAW invasion. Noteably, our model outcomes align most closely with those of du Plessis et al.^41^, Timilsena et al.^44^, and Wang et al.^45^ potentially due to their similarity in parameter values, except for SM3, Wet Stress, and Cold Stress. Cold Stress remains the most crucial factor limiting the extent of FAW overwintering areas in southern Europe, reflecting the model’s high sensitivity to this parameter. Given that we used a higher TTCS value than most CLIMEX studies on FAW, adjusting the THCS value proportionately produced a similar response in the study area as the aforementioned studies^44,45^.

A critical aspect of FAW risk in Europe is its migration capacity during the warmer months. It is well known that FAW flies long distances northwards during the corn season, from the southeast coast of the USA up to Canada, to exploit areas where the climate is seasonally suitable for its temporary persistence^68^. Based on the model projections, the climatic conditions in Europe that are favourable for ephemeral FAW populations extend to a large portion of the continent. These findings are consistent with previous research^41,44–46^ and are strongly in agreement with the work of Senay et al.^46^. Given that our model indicates suitable pockets for permanent establishment along the Mediterranean, European agriculture may be threatened by seasonal populations of FAW adults migrating across large swathes of the continent. The magnitude of these threats will depend on the density of the established FAW population, the proximity to the permanent population establishment zone, and the seasonal wind patterns^79^. Lastly, even though the projected area supporting FAW’s permanent establishment in Europe is relatively small, climate change will likely extend this zone northwards in Europe.

Our analysis of FAW migration patterns in its native range provides further evidence of the potential for its seasonal migration and associated impacts throughout Europe. An interesting finding is the right-skewed shape of the distribution of flight distances, implying that most transient occurrence records are concentrated at shorter distances from year-round source populations. This outcome resembles the findings on *Spodoptera eridania* reported in Weinberg et al.^79^. Another characteristic is the broad range of values of migration distances, suggesting the inherent unpredictability of individual annual FAW migratory patterns. Our analysis revealed that FAW may migrate over 2 000 km from overwintering areas with the 75th and 95th percentile distances reaching 1 079 km and 1 509 km, respectively. Formal expert knowledge elicited (EKE) estimates for FAW’s annual spread in Europe^81^ (1 178 km and 1 436 km, respectively), provide a relevant point of reference. Our study, therefore, demonstrates that a zone of approximately 1 000 km from overwintering areas may bear the highest risk of annual FAW migration, which provides further insight into where limited resources should be targeted to help farmers manage its impact.

Our results show that the introduction and establishment of FAW in Europe could severely impact grain maize production, with southern MSs such as Greece, Portugal, Italy, France, and Spain bearing the greatest risk. Gross margin losses account for a substantial proportion of the average baseline grain maize gross margins per hectare (469 €/ha across the 13 MSs) over the period 2010–2020, ranging from 17 to 30% under the moderate case scenario, and escalating to 65–116% in the worst-case scenario. Moreover, MSs with low average grain maize gross margins that have previously experienced unprofitable production (e.g., Slovenia in 2013 and 2014) may face this distressing situation more frequently due to FAW. The vulnerability of the southern MSs stems from their proximity to areas suitable for FAW establishment, and their relatively high baseline gross margins per ha for grain maize. When considering economic losses, major grain maize producers like France, Italy, and Spain are projected to be impacted the most, losing annually from 109 to 262 million €, under the worst-case scenario. This divergence between per-hectare gross margins and total losses of million € is primarily attributable to two factors. Firstly, scaling up the per-hectare gross margins to account for the total cultivated area in each MS provides a more comprehensive reflection of production volume. Secondly, geographical distance from the areas suitable for FAW establishment plays a critical role. Countries such as Poland and Hungary, though large grain maize producers are located further from areas of potential FAW establishment than France and Italy, thereby facing a lower relative risk of economic loss from FAW migration. Being further north, it will take FAW more generations to migrate there each year, and the probability of any impacts each year is likely decreased.

Our assessment of FAW’s direct economic impact on European grain maize production is based on the CLIMEX projections and observed migratory behaviour in its native range. While these results highlight the pest’s damage potential, it should be noted that they are subject to certain assumptions and shortcomings. We assumed that all FAW occurrence records outside areas of permanent establishment represent transient populations that dispersed naturally by flight. While sufficient evidence supports FAW’s capacity for such long-distance migration via flight^1,2,30,68^, this method does not account for other potential dispersal pathways, such as human-mediated ones. The potential extent of annual migration within the European continent was also estimated based on transient FAW populations observed in the USA and Canada, and assumed identical flying capacity in Europe. However, it could be argued that the migration patterns in Europe may differ due to various factors influencing the pest’s behaviour, such as prevailing wind patterns during the migration season^45^. Due to the uncertainty around FAW’s natural dispersal each year, and its periodic nature (annual recurrence), we did not employ a mechanistic spatiotemporal spread model where the pest disperses progressively. Instead, our study provides a quantitative baseline of what an imminent FAW invasion could mean for Europe without considering the temporal dimension of the invasion. Previous studies^43,45^ used the numerical trajectory HYSPLIT model to simulate FAW migration from North Africa into Europe. Employing the same method, it would be interesting to use our modelled suitable areas in Europe as FAW’s source locations to explore potential migratory patterns to the northern parts of the continent. However, the steady accumulation of false positive errors in mechanistic spread models with each timestep limits the value of such methods for informing biological invasion processes in bioeconomic impact models^87^.

The potential direct economic damage was calculated using the average grain maize gross margin by MS. However, gross margins vary within and between countries. Moreover, due to data availability, not all EU MSs were included in the analysis, nor were all the pest’s main host plants. Consequently, the reported impacts most likely underestimate the potential damage of FAW. Nonetheless, these results provide valuable new insights, even for MSs that were not included in the assessment. Finally, the calculations were performed under a “no-control” scenario, implying that these loss estimates are the highest they can be. Clearly, one should expect that farmers would respond to prevent or mitigate the impacts of FAW. However, forecasting potential economic impacts without control provides a baseline upon which future investigations can consider the costs and benefits of management options.

Although this study focuses on the potential economic impact of FAW on grain maize, it should be noted that the pest poses additional risks to a range of other economically important crops cultivated in Europe, including rice, sorghum, soybean, and cotton^2,6,43^. Our projections are based on historical climate, and we did not simulate any future climate change scenarios as equivalent datasets were not yet available. However, based on previous studies, a northward expansion of climatically suitable areas is likely under future climate change scenarios, potentially shifting the current boundaries of both permanent and transient FAW populations^44,49^. Lastly, while CLIMEX is used to estimate species’ climate suitability in a given location, host plant availability remains a critical factor influencing establishment success and, therefore, damage potential^2,44,49,54,57,79,88^. However, for highly polyphagous species such as FAW, host plant availability is generally not a limiting factor for establishment. Future work on FAW could benefit from incorporating spatial host distribution data on various crop species, alongside data on future climate change scenarios.

Our study lays the groundwork for future research on the potential economic damage of FAW in Europe and emphasizes the need for effective management. While supporting control efforts in North Africa and the Middle East could help reduce the risk of FAW natural dispersal into Europe^43^, recent detections of FAW adults in the Mediterranean, especially in Greece^38,40,86^ suggest that the establishment is already underway. Given FAW’s rapid spread and migratory capacity, prevention alone was always unlikely to be sufficient. Control efforts should also focus on the localised early detection and advice to farmers, and providing them with education packages and suitable, cost-effective, and environmentally safe tools to limit the impacts of FAW^88,89^. Moreover, since our model suggests that southern Europe hosts several pockets of climatic suitability for permanent FAW population establishment, and that Mediterranean MSs may face the greatest impacts, coordinated biosecurity measures should be prioritized in these regions.

## Electronic supplementary material

Below is the link to the electronic supplementary material.

Supplementary Material 1

Supplementary Material 2

## Data Availability

Data is provided within the manuscript or supplementary information files.The occurrence data used in this study are openly available and attached in the “Supplementary Information” section. Additionally, all the references/links for the economic data are provided in the text.
