# Supplementary material for: Migration extent and potential economic impact of the fall armyworm in Europe
Source: Sci Rep. 2025 May 19;15:17405. doi: 10.1038/s41598-025-02595-7 (PMC12089574; doi:10.1038/s41598-025-02595-7)
Supplement: Supplementary file 1 — Supplementary Material 1 [file 41598_2025_2595_MOESM1_ESM.docx]

Migration extent and potential economic impact of the fall armyworm in Europe

## Supplementary Information

Stelios Kartakis^1^, Kiran J. Horrocks^2^, Kutay Cingiz^1^, Darren J. Kriticos^3,4^, Justus Wesseler^1^

^1^ Agricultural Economics and Rural Policy Group, Wageningen University, The Netherlands

^2^ Biosafety Research Group, Agroscope, Switzerland

^3^ Cervantes Agritech Pty Limited, Canberra, ACT, Australia

^4^ Applied Biosciences, Macquarie University, Sydney, NSW, Australia

*Correspondence: [stelios.kartakis@wur.nl](mailto:stelios.kartakis@wur.nl)

The following supporting information is provided:

[Figure S1. The number of interceptions of imported commodities (plants and other objects) into the EU and Switzerland due to the detection of Spodoptera frugiperda from 2011 to 2019. (Source: author’s representation based on EUROPHYT https://food.ec.europa.eu/plants/plant-health-and-biosecurity/europhyt/interceptions_en#annual_interceptions) 4](#_Toc190419806)

[Figure S2. Global climatic suitability of Spodoptera frugiperda modeled using the Compare Locations module in CLIMEX v4.0.2.0. ran with 30-year average climatic data centered on 1975 (World_1975_V1.2) under (a) rainfed conditions and (b) a composite irrigation scenario (2.5 mm day^-1^ applied as top-up). The Ecoclimatic Index (EI) and Growth Index (GI_A_) are the outputs of the parameters used in Table 1. The EI gradient (yellow-red) represents areas suitable for all year-round FAW population establishment. The GI_A_ gradient (light blue-dark blue) depicts areas suitable for seasonal population growth and migration. The figure was created with QGIS version 3.36.2 (https://qgis.org/). 7](#_Toc190419807)

[Figure S3. Projected climatic suitability of Spodoptera frugiperda (zoomed in on Europe) ran with 30-year average climatic data centered on 1975 (World_1975_V1.2) under (a) rainfed conditions (b) a composite irrigation scenario (2.5 mm day^-1^ applied as top-up), and ran with climate data 30-year average climatic data centered on 1995 (CM_TC10_1995_v1) under (c) rainfed conditions (d) a composite irrigation scenario (2.5 mm day^-1^ applied as top-up). The figure was created with QGIS version 3.36.2 (https://qgis.org/ 8](#_Toc190419808)

Figure S4. Global maps of climatic suitability projections for Spodoptera frugiperda using CLIMEX parameter values from different studies. (a) Ramirez-Cabral et al.^3^, (b) du Plessis et al.^4^, (c) Timilsena et al.^5^, (d) Senay et al.^6^, (e) Wang et al.^7^, and (f) present study. The climatic suitability was modeled for all cases using the “Compare Location” module in CLIMEX v4.0.2.0. ran with 30-year average climatic data centered on 1975 (World_1975_V1.2) and an irrigation scenario (2.5 mm day^-1^ applied as top-up), even if it was not applied in the original study. The classification system remains the same as the one used in the main text, to make the outputs comparable (unsuitable for EI=0, marginal for 0<EI≤5, moderate for 5<EI≤15, suitable for 15<EI≤30 and optimal for EI>30). The figure was created with QGIS version 3.36.2 (https://qgis.org/). 9

Figure S5. Projected climatic suitability of Spodoptera frugiperda using CLIMEX parameter values from different studies (zoomed in on Europe). (a) Ramirez-Cabral et al.^3^, (b) du Plessis et al.^4^, (c) Timilsena et al.^5^, (d) Senay et al.^6^, (e) Wang et al.^7^, and (f) present study. The climatic suitability was modeled for all cases using the “Compare Location” module in CLIMEX v4.0.2.0. ran with 30-year average climatic data centered on 1975 (World_1975_V1.2) and an irrigation scenario (2.5 mm day^-1^ applied as top-up), even if it was not applied in the original study. The classification system remains the same as the one used in the main text to make the outputs comparable (unsuitable for EI=0, marginal for 0<EI≤5, moderate for 5<EI≤15, suitable for 15<EI≤30 and optimal for EI>30). The figure was created with QGIS version 3.36.2 (https://qgis.org/). 10

[Figure S6. Distribution of Spodoptera frugiperda migration distances (in km) from the area of permanent establishment (EI>0) in the USA and Canada, using the data subset (n = 1831). The 25^th^, 50^th^, 75^th^, and 95^th^ percentiles of the migration distance distribution are indicated. The figure was created with ggplot2 package in R Studio version 4.3.3. (https://www.r-project.org/) 11](#_Toc190419811)

[Figure S7. The fitted empirical cumulative distribution function is based on Spodoptera frugiperda migration distances from the permanent establishment (EI>0) area in the USA and Canada. The curve illustrates the cumulative probability that the pest would fly a certain distance away from EI>0. The figure was created with ggplot2 package in R Studio version 4.3.3. (https://www.r-project.org/) 12](#_Toc190419812)

[Figure S8. The probability of Spodoptera frugiperda annual presence in 13 EU Member States, based on the distance of the centroid of each Member State to the closest projected area of permanent establishment (EI>0). The assigned probability for each Member State is derived from the fitted ECDF curve based on the historical migration data from the USA and Canada. The figure was created with ggplot2 and ggrepel packages in R Studio version 4.3.3. (https://www.r-project.org/) 13](#_Toc190419813)

[Figure S9. Projected climatic suitability of Spodoptera frugiperda in Europe modeled using the Compare Locations module in CLIMEX v4.1.1.0 ran with 30-year average climatic data centered on 1995 (CM_TC10_1995_v1). FAW dispersal frequency zones are depicted using cross-hatching buffer zones and are based on FAW’s migratory patterns in the USA and Canada. The diagonal hatching buffer zone extends to a 1079 km distance from the area of permanent establishment (75^th^ percentile). The buffer zone was obtained, using the “distance from nearest hub” function in QGIS version 3.36.2 (https://qgis.org/). 14](#_Toc190419814)

[Figure S10. The annual direct economic impacts (in €/ha) of Spodoptera frugiperda on grain maize production in different EU Member States. Green, yellow, and red bars represent the annual gross margin loss under the best, moderate, and worst-case scenarios, respectively. The best-case scenario corresponds to the 2.25^th^ percentile of the Member-State-specific yield loss distribution. The moderate and worst-case scenarios represent the 50^th^ and 97.5^th^ percentile of the yield loss distribution, respectively. The figure was created with ggplot2 package in R Studio version 4.3.3. (https://www.r-project.org/) 15](#_Toc190419815)

[Figure S11. The annual direct economic impacts (in million €) of Spodoptera frugiperda on grain maize production in different EU Member States. Green, yellow, and red bars represent the annual gross margin loss under the best, moderate, and worst-case scenarios, respectively. The best-case scenario corresponds to the 2.25^th^ percentile of the Member-State-specific yield loss distribution. The moderate and worst-case scenarios represent the 50^th^ and 97.5^th^ percentile of the yield loss distribution, respectively. The figure was created with ggplot2 package in R Studio version 4.3.3. (https://www.r-project.org/) 16](#_Toc190419816)

[Table S1. Justification/Reasoning for each yield loss scenario on grain maize in the EU, based on formal EKE data for Spodoptera frugiperda. Source: EFSA, et al., (2019)^2^ https://doi.org/10.5281/zenodo.2789779 17](#_Toc182570434)

[Table S2. Expert elicited parameter for potential yield impacts by Spodoptera frugiperda on grain maize per EU Member State. Source: EFSA, et al., (2019)^2^ https://doi.org/10.5281/zenodo.2789779 18](#_Toc182570435)

[Table S3. Average annual grain maize gross margins (€/ha) and gross margin relative decrease (%) due to Spodoptera frugiperda in Europe, under different yield loss scenarios. 19](#_Toc182570436)

**Figure S1.** The number of interceptions of imported commodities (plants and other objects) into the EU and Switzerland due to the detection of Spodoptera frugiperda from 2011 to 2019. (Source: author’s representation based on EUROPHYT <https://food.ec.europa.eu/plants/plant-health-and-biosecurity/europhyt/interceptions_en#annual_interceptions>)

## Global potential distribution of *Spodoptera frugiperda* under current climatic conditions

### Native range

The output of the CLIMEX model accords with FAW's known native year-round distribution (**Fig. 2**). FAW originates from South and Central America, the Caribbean, and the southern part of Texas and Florida in the USA. The EI values in these regions exceed 30, indicating optimal climatic suitability under both rainfed and irrigation scenarios. Additionally, the vast majority of the transient FAW occurrence records falls within the area with GI values above 15 (suitable to optimal), while the remaining approximately 5% of the records are in areas that exhibit at least moderate suitability (5<GI≤15). The projected suitable range for FAW’s ephemeral populations extends south to northern Argentina and north to the eastern USA and Canada, encompassing regions with humid-subtropical and humid-continental climates.

### Invaded range

In Africa, most of the collected FAW occurrence records fall within areas with optimal climatic conditions (EI>30), under both rainfed and irrigation scenarios. The model also closely accords with the distributional extremes of FAW’s year-round distribution, which is particularly visible in West Africa. Under the rainfed scenario, the model is largely in line with the year-round distribution of FAW, predicting population establishment that extends from coastal South Africa, up to the area below the Sahel zone, excluding the south of Namibia, Botswana, and Lesotho,. The hot desert climates in Djibouti and the western parts of Somalia and Eritrea were not predicted to support permanent populations, mainly attributed to dry stress. However, the model did not capture occurrence points along the Nile River Basin in Egypt under the rainfed scenario. When the irrigation scenario was applied, the model accorded with most of these occurrence points, though some remained outside of predicted climatic suitability. Similarly, occurrence records in irrigated areas of Central Niger fall within predicted climatic suitability under the irrigation scenario. Permanent FAW populations could also be supported in irrigated areas of Djibouti, Somalia, and Eritrea, where the climate was not suitable before.

The model assigns high EI values with FAW occurrence records in southeast Asian countries, most of India, southern China, and southern Nepal, where year-round populations occur. In particular, the model closely accords with the northern limits of year-round fall armyworm populations in southern China. A difference between the rainfed and irrigation scenario, aside from the permanent FAW establishment extent, is the inclusion of few occurrence records in Pakistan. Records in China, South Korea, and Japan that represent seasonal migratory FAW populations, fall within areas with high predicted GI values. However, moderate GI values were predicted further north of known seasonal occurrence records. Migratory behavior is observed in these areas, with occurrence records outside the EI>0 area but within the GI>30 zones. This behavior is apparent, especially on the coast of South Korea and Japan, as well as inland Chinese provinces, such as Guizhou, Jiangxi, and Yunnan.

Tropical and subtropical areas in Oceania are predicted to be highly suitable for FAW establishment. Specifically, CLIMEX projected optimal suitability in the Solomon Islands, Vanuatu, New Caledonia, Norfolk Island, Badu Island, and Papua New Guinea. In Australia, the model agrees with the distribution of known year-round FAW populations in the tropical and subtropical areas of the Northern Territory and Queensland, as well as Western Australia. The remainder of Australia was predicted to be unsuitable, though when irrigation is applied, more inland areas of the Northern Territory are predicted to support permanent FAW population establishment. Furthermore, climatic conditions appear to be suitable for FAW establishment in the Northland and Auckland regions of New Zealand.

The model predicts that the Mediterranean coast of Europe and North Africa can support permanent FAW establishment under both rainfed and irrigation scenarios. In particular, large areas of northern Egypt, northern Libya, Tunisia, Portugal, Sicily, and the Greek islands are predicted to be climatically suitable. The occurrence records on mainland Greece (Laconia and Eastern Attica) are in areas predicted to be suitable for permanent FAW establishment, whereas the occurrence record in Romania is not. However, the majority of Europe appears to be suitable for FAW migratory populations exhibiting moderate to suitable GI values. This seasonal occurrence of migratory FAW populations may expand far beyond the limited area predicted to be suitable for year-round population persistence.


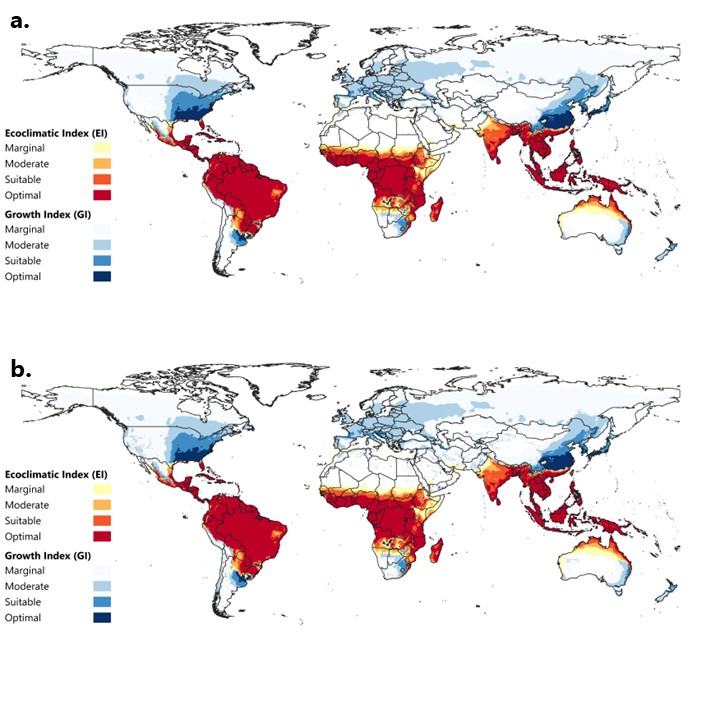


**Figure S2**. Global climatic suitability of Spodoptera frugiperda modeled using the Compare Locations module in CLIMEX v4.0.2.0. ran with 30-year average climatic data centered on 1975 (World_1975_V1.2) under **(a)** rainfed conditions and **(b)** a composite irrigation scenario (2.5 mm day^-1^ applied as top-up). The Ecoclimatic Index (EI) and Growth Index (GI_A_) are the outputs of the parameters used in **Table 1**. The EI gradient (yellow-red) represents areas suitable for all year-round FAW population establishment. The GI_A_ gradient (light blue-dark blue) depicts areas suitable for seasonal population growth and migration. The figure was created with QGIS version 3.36.2 (<https://qgis.org/>).


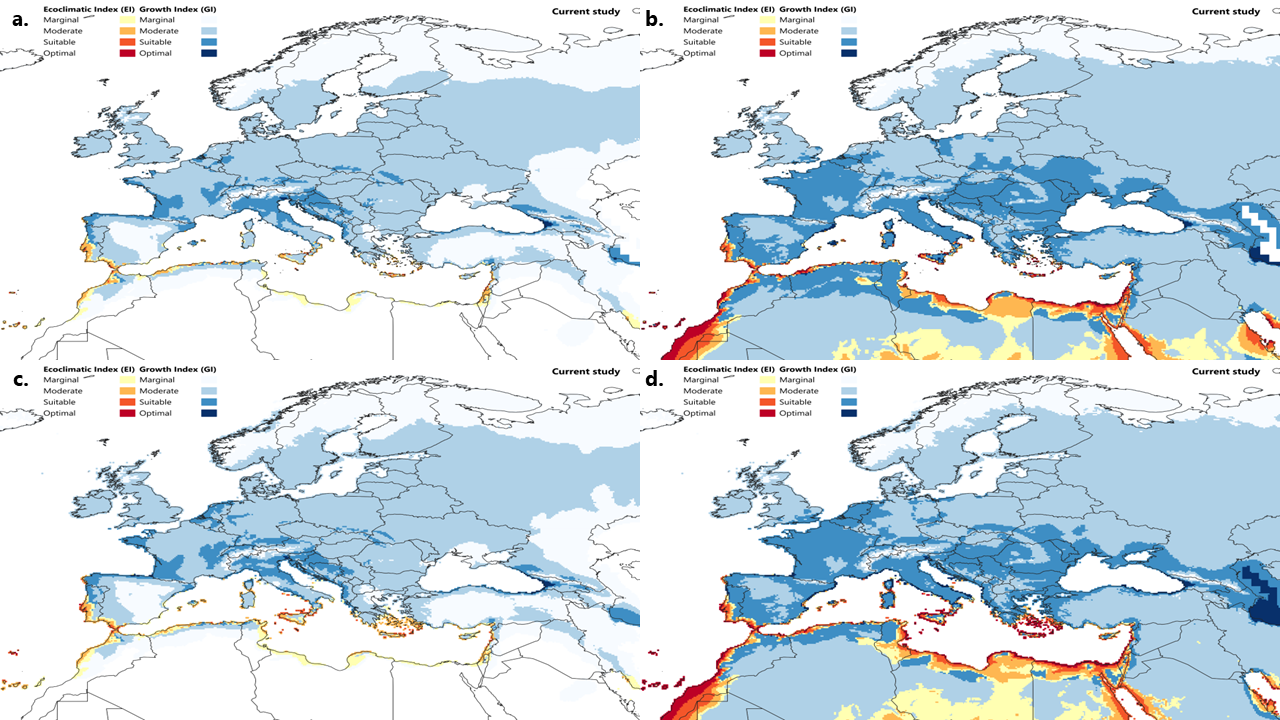


**Figure S3.** Projected climatic suitability of Spodoptera frugiperda (zoomed in on Europe) ran with 30-year average climatic data centered on 1975 (World_1975_V1.2) under **(a)** rainfed conditions **(b)** an irrigation scenario (2.5 mm day^-1^ applied as top-up), and ran with climate data 30-year average climatic data centered on 1995 (CM_TC10_1995_v1) under **(c)** rainfed conditions **(d)** a composite irrigation scenario (2.5 mm day^-1^ applied as top-up). The figure was created with QGIS version 3.36.2 (<https://qgis.org/>).


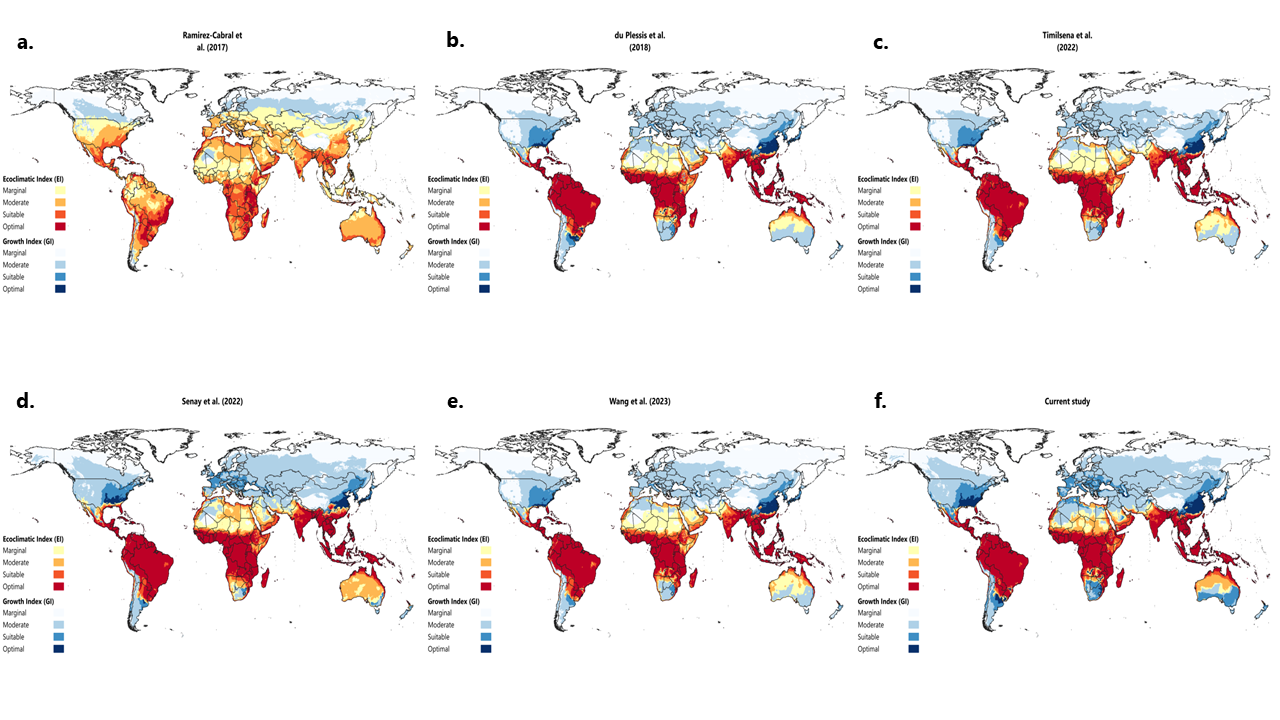


**Figure S4.** Global maps of climatic suitability projections for Spodoptera frugiperda using CLIMEX parameter values from different studies. **(a)** Ramirez-Cabral et al.^3^, **(b)** du Plessis et al.^4^, **(c)** Timilsena et al.^5^, **(d)** Senay et al.^6^, **(e)** Wang et al.^7^, and **(f)** present study. The climatic suitability was modeled for all cases using the “Compare Location” module in CLIMEX v4.0.2.0. ran with 30-year average climatic data centered on 1975 (World_1975_V1.2) and an irrigation scenario (2.5 mm day^-1^ applied as top-up), even if it was not applied in the original study. The classification system remains the same as the one used in the main text, to make the outputs comparable (unsuitable for EI=0, marginal for 0<EI≤5, moderate for 5<EI≤15, suitable for 15<EI≤30 and optimal for EI>30). The figure was created with QGIS version 3.36.2 (<https://qgis.org/>).


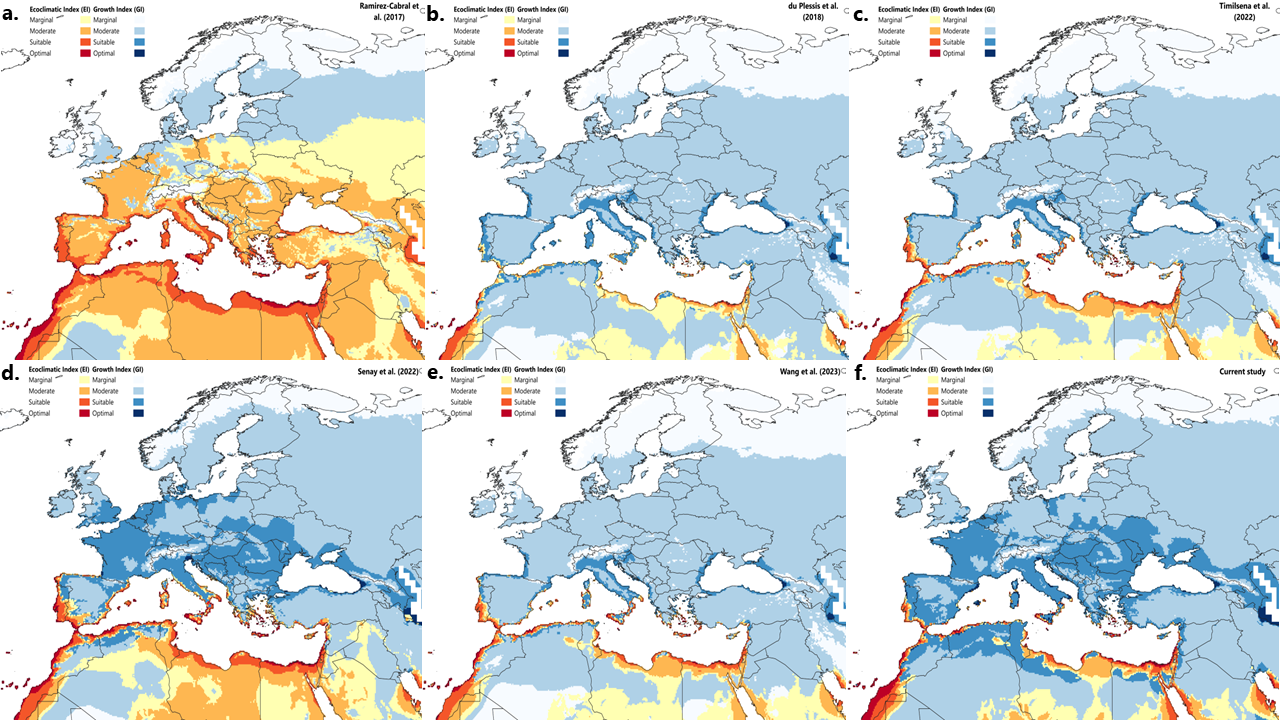


**Figure S5.** Projected climatic suitability of Spodoptera frugiperda using CLIMEX parameter values from different studies (zoomed in on Europe). **(a)** Ramirez-Cabral et al.^3^, **(b)** du Plessis et al.^4^, **(c)** Timilsena et al.^5^, **(d)** Senay et al.^6^, **(e)** Wang et al.^7^, and **(f)** present study. The climatic suitability was modeled for all cases using the “Compare Location” module in CLIMEX v4.0.2.0. ran with 30-year average climatic data centered on 1975 (World_1975_V1.2) and an irrigation scenario (2.5 mm day^-1^ applied as top-up), even if it was not applied in the original study. The classification system remains the same as the one used in the main text to make the outputs comparable (unsuitable for EI=0, marginal for 0<EI≤5, moderate for 5<EI≤15, suitable for 15<EI≤30 and optimal for EI>30). The figure was created with QGIS version 3.36.2 (<https://qgis.org/>).


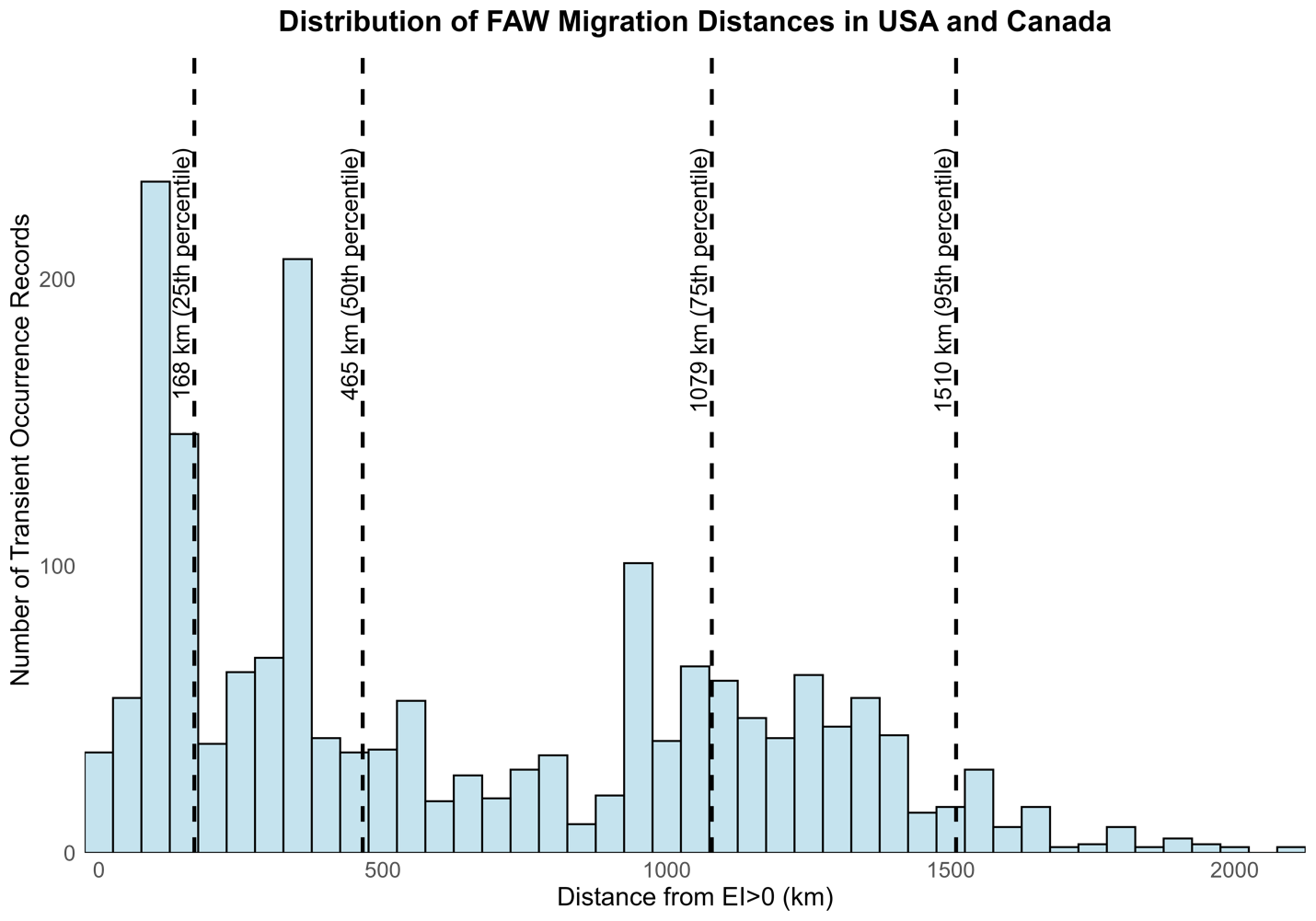


**Figure S6.** Distribution of Spodoptera frugiperda migration distances (in km) from the area of permanent establishment (EI>0) in the USA and Canada, using the data subset (n = 1831). The 25^th^, 50^th^, 75^th^, and 95^th^ percentiles of the migration distance distribution are indicated. The figure was created with ggplot2 package in R Studio version 4.3.3. (<https://www.r-project.org/>).


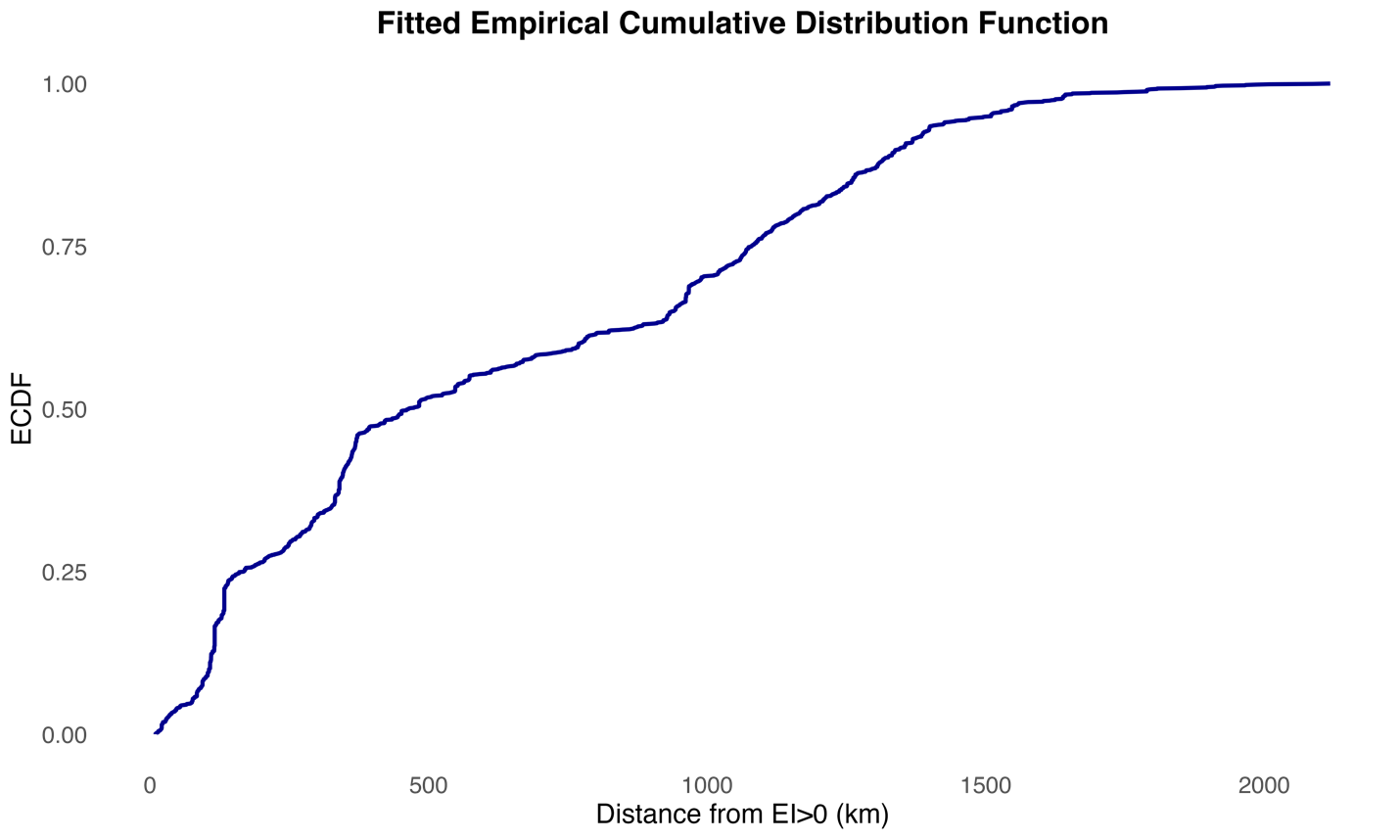


**Figure S7.** The fitted empirical cumulative distribution function is based on Spodoptera frugiperda migration distances from the permanent establishment (EI>0) area in the USA and Canada. The curve illustrates the cumulative probability that the pest would fly a certain distance away from EI>0. The figure was created with ggplot2 package in R Studio version 4.3.3. (<https://www.r-project.org/>).


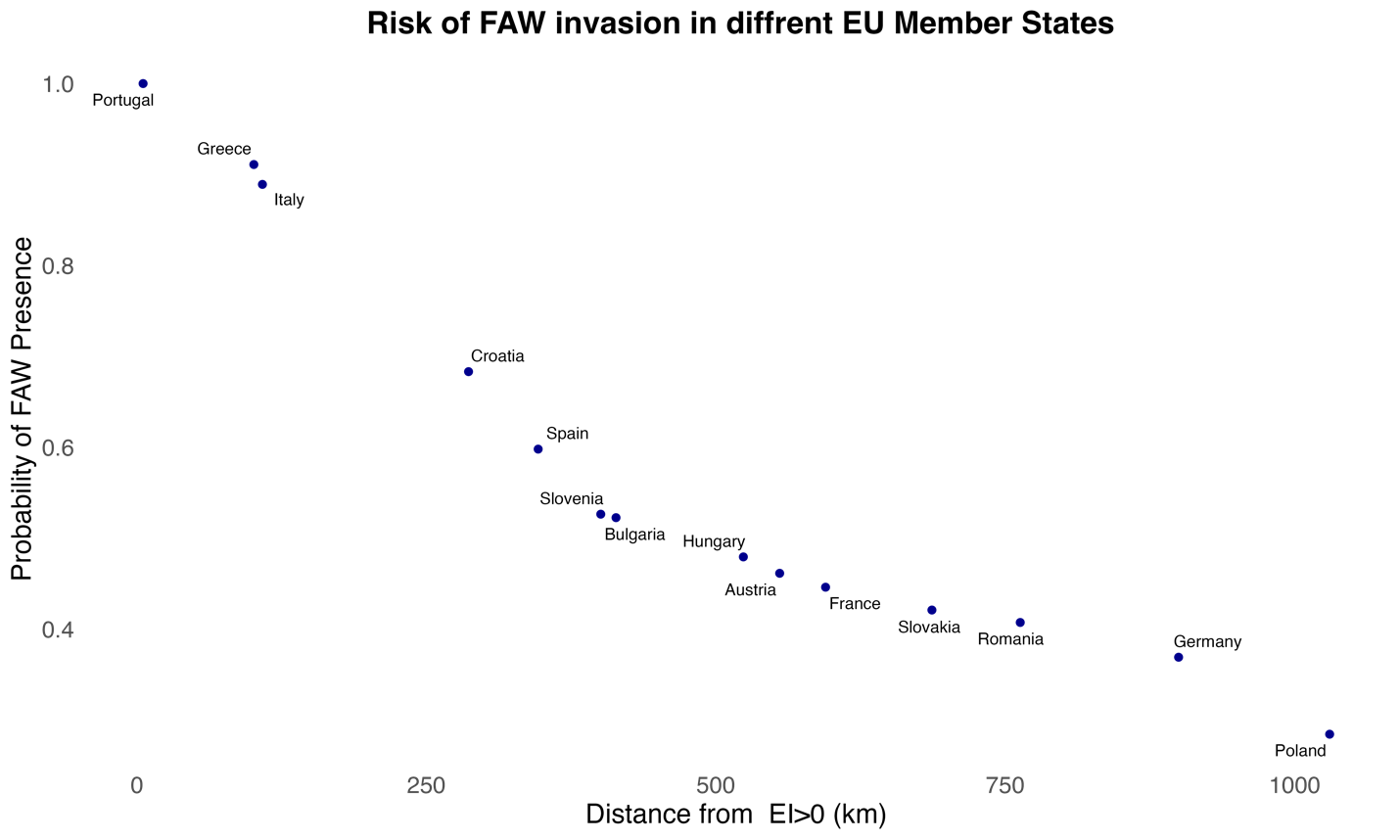


**Figure S8.** The probability of Spodoptera frugiperda annual presence in 13 EU Member States, based on the distance of the centroid of each Member State to the closest projected area of permanent establishment (EI>0). The assigned probability for each Member State is derived from the fitted ECDF curve based on the historical migration data from the USA and Canada. The figure was created with ggplot2 and ggrepel packages in R Studio version 4.3.3. (<https://www.r-project.org/>).


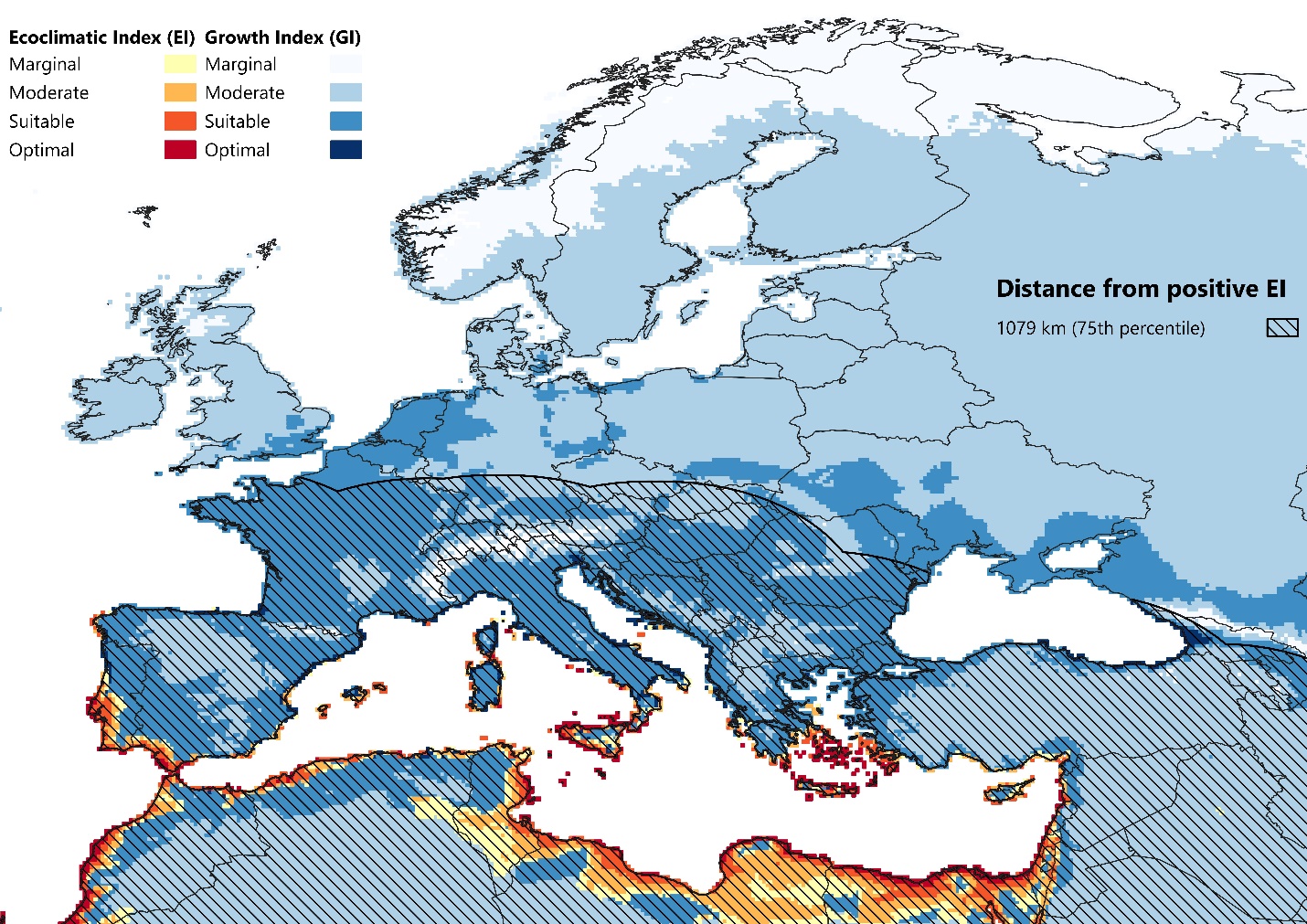


**Figure S9.** Projected climatic suitability of Spodoptera frugiperda in Europe modeled using the Compare Locations module in CLIMEX v4.1.1.0 ran with 30-year average climatic data centered on 1995 (CM_TC10_1995_v1). FAW dispersal frequency zones are depicted using cross-hatching buffer zones and are based on FAW’s migratory patterns in the USA and Canada. The diagonal hatching buffer zone extends to a 1079 km distance from the area of permanent establishment (75^th^ percentile). The buffer zone was obtained, using the “distance from nearest hub” function in QGIS version 3.36.2 (<https://qgis.org/>).


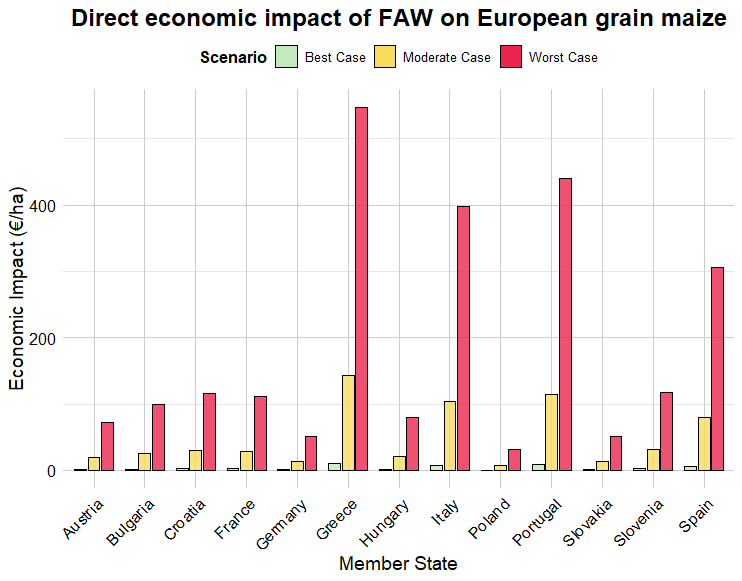


**Figure S10.** The annual direct economic impacts (in €/ha) of Spodoptera frugiperda on grain maize production in different EU Member States. Green, yellow, and red bars represent the annual gross margin loss under the best, moderate, and worst-case scenarios, respectively. The best-case scenario corresponds to the 2.25^th^ percentile of the Member-State-specific yield loss distribution. The moderate and worst-case scenarios represent the 50^th^ and 97.5^th^ percentile of the yield loss distribution, respectively. The figure was created with ggplot2 package in R Studio version 4.3.3. (<https://www.r-project.org/>).


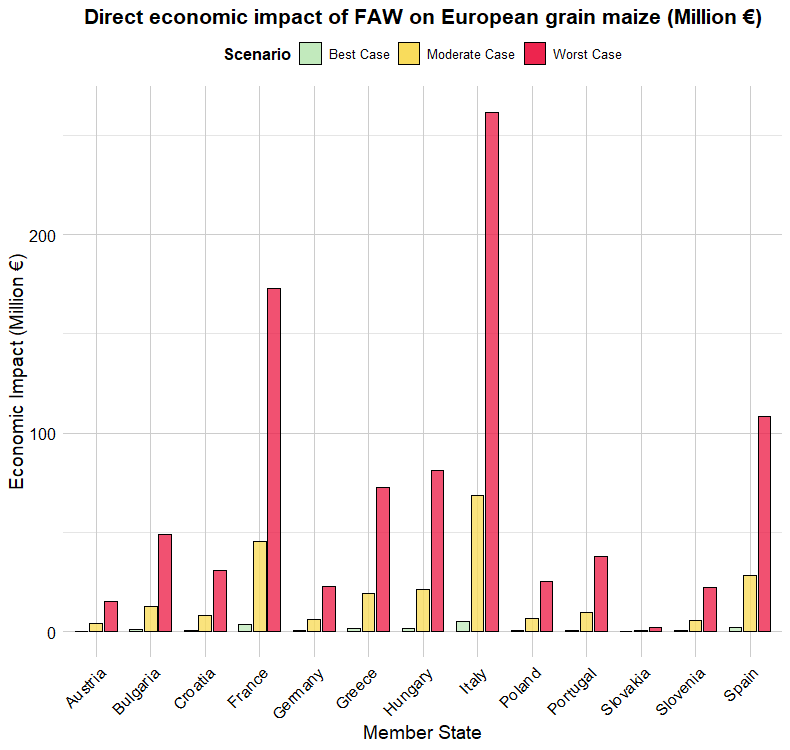


**Figure S11.** The annual direct economic impacts (in million €) of Spodoptera frugiperda on grain maize production in different EU Member States. Green, yellow, and red bars represent the annual gross margin loss under the best, moderate, and worst-case scenarios, respectively. The best-case scenario corresponds to the 2.25^th^ percentile of the Member-State-specific yield loss distribution. The moderate and worst-case scenarios represent the 50^th^ and 97.5^th^ percentile of the yield loss distribution, respectively. The figure was created with ggplot2 package in R Studio version 4.3.3. (<https://www.r-project.org/>).

**Table S1.** Justification/Reasoning for each yield loss scenario on grain maize in the EU, based on formal EKE data for Spodoptera frugiperda. Source: EFSA, et al., (2019)^2^ <https://doi.org/10.5281/zenodo.2789779>

| Yield loss scenario | | |
| --- | --- | --- |
| Best case (2.5^th^ percentile) | **Moderate case (50^th^ percentile)** | **Worst case (97.5^th^ percentile)** |
| Current control measures are effective against FAW | Current control methods are effective against FAW | FAW reaches most of the EU’s grain maize production area at the most susceptible stage |
| Treatments are expected to be applied more frequently and with better timing | Some infestation of the cobs may not affect yield substantially (unlike with sweet corn) | High population abundance affects both leaves and cobs |
| The rejection rate is not as high as other host crops (e.g., sweet corn) | The climate in the EU is not as suitable as in Africa | Only one generation attacks each crop: the following generation flies to the next crop |
| It is less likely that FAW attacks the cob since infestations begin with the leaves and do not necessarily reach the cob | Infestation is more likely to affect the leaves than the cobs | Treatments that are not targeted at FAW may not be fully effective (e.g., poor timing) |

**Table S2.** Expert elicited parameter for potential yield impacts by Spodoptera frugiperda on grain maize per EU Member State. Source: EFSA, et al., (2019)^2^ <https://doi.org/10.5281/zenodo.2789779>

| Member State | Yield loss (%) distribution percentile | | | | |
| --- | --- | --- | --- | --- | --- |
|  | **2.25^th^** | **25^th^** | **50^th^** | **75^th^** | **97.5^th^** |
| Austria | 0.22 | 1.40 | 2.88 | 5.06 | 11.02 |
| Bulgaria | 0.406 | 2.57 | 5.27 | 9.27 | 20.16 |
| Germany | 0.20 | 1.27 | 2.61 | 4.59 | 9.98 |
| Greece | 0.53 | 3.40 | 6.99 | 12.28 | 26.71 |
| Spain | 0.50 | 3.17 | 6.52 | 11.45 | 24.92 |
| France | 0.29 | 1.89 | 3.88 | 6.81 | 14.82 |
| Croatia | 0.41 | 2.61 | 5.36 | 9.41 | 20.48 |
| Hungary | 0.30 | 1.93 | 3.96 | 6.96 | 15.139 |
| Italy | 0.45 | 2.87 | 5.90 | 10.37 | 22.56 |
| Poland | 0.20 | 1.26 | 2.60 | 4.57 | 9.94 |
| Portugal | 0.46 | 2.93 | 6.01 | 10.56 | 22.98 |
| Slovenia | 0.36 | 2.28 | 4.68 | 8.22 | 17.88 |
| Slovakia | 0.24 | 1.52 | 3.12 | 5.48 | 11.92 |

**Table S3.** Average annual grain maize gross margins (€/ha) and gross margin relative decrease (%) due to Spodoptera frugiperda in Europe, under different yield loss scenarios.

| Member State | Baseline | Best Case | | Moderate Case | | Worst Case | |
| --- | --- | --- | --- | --- | --- | --- | --- |
|  | (€/ha) | (€/ha) | (%) | (€/ha) | (%) | (€/ha) | (%) |
| Austria | 373 | 372 | 0.4 | 354 | 5.1 | 300 | 19.5 |
| Bulgaria | 446 | 444 | 0.5 | 420 | 5.9 | 346 | 22.5 |
| Croatia | 196 | 194 | 1.2 | 166 | 15.5 | 80 | 59.4 |
| France | 375 | 373 | 0.6 | 346 | 7.7 | 264 | 29.6 |
| Germany | 343 | 342 | 0.3 | 330 | 3.9 | 293 | 14.8 |
| Greece | 766 | 755 | 1.4 | 623 | 18.7 | 220 | 71.3 |
| Hungary | 417 | 415 | 0.4 | 396 | 5.0 | 337 | 19.2 |
| Italy | 756 | 748 | 1.1 | 652 | 13.8 | 357 | 52.7 |
| Poland | 408 | 408 | 0.2 | 400 | 2.0 | 377 | 7.6 |
| Portugal | 760 | 752 | 1.2 | 645 | 15.1 | 321 | 57.8 |
| Slovakia | 189 | 188 | 0.6 | 175 | 7.2 | 137 | 27.4 |
| Slovenia | 81 | 78 | 2.9 | 50 | 38.3 | -37 | 146.2 |
| Spain | 985 | 979 | 0.6 | 905 | 8.1 | 679 | 31.1 |
| *Average* | 469 | 465 | 0.4 | 420 | 5.1 | 283 | 19.5 |

# References

1. Huang, Y. *et al.* Mapping the Spatio-Temporal Distribution of Fall Armyworm in China by Coupling Multi-Factors. *Remote Sens (Basel)* **14**, 4415 (2022).

2. EFSA *et al.* Spodoptera frugiperda ̶ Pest Report and Datasheet to support ranking of EU candidate priority pests [Data set]. *Zenodo* Preprint at https://doi.org/https://doi.org/10.5281/zenodo.2789779 (2019).

3. Ramirez-Cabral, N. Y. Z., Kumar, L. & Shabani, F. Future climate scenarios project a decrease in the risk of fall armyworm outbreaks. *J Agric Sci* **155**, 1219–1238 (2017).

4. du Plessis, H., van den Berg, J., Ota, N. & Kriticos, D. J. Spodoptera frugiperda (Fall Armyworm). *Pest Geography* (2018).

5. Paudel Timilsena, B. *et al.* Potential distribution of fall armyworm in Africa and beyond, considering climate change and irrigation patterns. *Sci Rep* **12**, 539 (2022).

6. Senay, S. D., Pardey, P. G., Chai, Y., Doughty, L. & Day, R. Fall armyworm from a maize multi-peril pest risk perspective. *Frontiers in Insect Science* **2**, 971396 (2022).

7. Wang, J. *et al.* Migration risk of fall armyworm (Spodoptera frugiperda) from North Africa to Southern Europe. *Front Plant Sci* **14**, 1141470 (2023).
